# SEVs-mediated miR-6750 transfer inhibits pre-metastatic niche formation in nasopharyngeal carcinoma by targeting M6PR

**DOI:** 10.1038/s41420-022-01262-4

**Published:** 2023-01-06

**Authors:** Caiming Zhang, Wenhui Chen, Si Pan, Siyu Zhang, Haijing Xie, Zixiang Zhang, Wei Lei, Lili Bao, Yiwen You

**Affiliations:** 1 Department of Otorhinolaryngology Head and Neck Surgery, Affiliated Hospital of Nantong University, Nantong, China; 2 Institute of Otolaryngology Head and Neck Surgery, Affiliated Hospital of Nantong University, Nantong, China

**Keywords:** Cancer microenvironment, Head and neck cancer

## Abstract

Reliable detection of circulating small extracellular vesicles (SEVs) and their miRNA cargo has been needed to develop potential specific non-invasive diagnostic and therapeutic marker for cancer metastasis. Here, we detected miR-6750, the precise molecular function of which was largely unknown, was significantly enriched in serum-SEVs from normal volunteers vs. patients with nasopharyngeal carcinoma (NPC). And we determined that miR-6750-SEVs attenuated NPC metastasis. Subsequently, miR-6750-SEVs was proven to inhibit angiogenesis and activate macrophage toward to M1 phenotype to inhibit pre-metastatic niche formation. After analyzing the expression level of miR-6750 in NPC cells, HUVECs and macrophage, we found that once miR-6750 level in NPC cells was close to or higher than normal nasopharyngeal epithelial cells (NP69), miR-6750-SEVs would be transferred from NPC cells to macrophage and then to HUVECs to modulate metastatic niche. Moreover, in vitro assays and BALB/c mouse tumor models revealed that miR-6750 directly targeted mannose 6-phosphate receptor (M6PR). Taken together, our findings revealed that miR-6750-M6PR axis can mediate NPC metastasis by remodeling tumor microenvironment (TME) via SEVs, which give novel sights to pathogenesis of NPC.

## Introduction

Nasopharyngeal carcinoma (NPC), arising from nasopharyngeal mucosal lining, is the most common cancer in the head and neck. It has a unique geographical distribution, predominant in southern China and southeast Asia [1]. With the use of intensity-modulated radiotherapy (IMRT) and combined chemoradiotherapy, the prognosis of NPC patients has improved substantially [2, 3]. However, even though patients undergoing radical treatment, 30–40%, eventually develop distant metastasis [4]. And, distant metastasis is still the main reason for treatment failure [5]. Therefore, advances in identifying predictive metastatic biomarkers and clarifying underlying mechanisms are essential.

Small extracellular vesicles (SEVs) are extracellular nanovesicles, with an average diameter of 30–150 nanometers [6, 7]. Initially using the term “exosomes” by the international scientific community, they were later redefined as “SEVs” by the International Society for Extracellular Vesicles (ISEV) [8]. All cells release SEV for normal physiology or acquired abnormalities, including tumor initiation, metastasis and therapy-resistance [9–11]. Similar to their cell of origin, they carry diverse constituents, including proteins, RNA, DNA, glycans, lipids, and metabolites [12, 13]. Niche is defined as the concept of an environment in a secondary organ and tissue that can be conducive to the metastasis of a primary tumor, including immunosuppression, inflammation, angiogenesis/vascular permeability, lymphangiogenesis, organotropism, and reprogramming [14, 15]. Advanced research showed that cell-by-cell interaction by SEV signaling leads to pre-metastatic niche construction, which subsequently results in tumor metastasis [16–18].

MicroRNAs (miRNAs) are a class of conserved small non-coding RNAs, which functions as important post-transcriptional regulators by binding to the 3′-untranslated region (3′-UTR) of target messenger RNAs (mRNAs) [19]. MiRNAs have been reported to regulate more than one-third of human mRNAs, which participate in regulating a number of biological processes [20, 21]. Amit et al. found that loss of TP53 leads to adrenergic transdiferentiation of nerves in OSCC through downregulation of the miR-34a level [22]. Flemming et al. showed that Dsg2 down-regulated both cellular and SEVs-loaded miR-146a in head and neck squamous cell carcinoma (HNSCC) to modulates tumour development and progression [23]. Interestingly, in our previous study, we have successfully isolated SEVs miRNAs and showed that metastasis-associated miR-23a from NPC-derived exosomes plays an important role in mediating angiogenesis by targeting TSGA10 [24]. Thus, a specific SEVs miRNA signature may reflect environment, responsible for NPC metastasis, which will further lead to developing more efficient SEVs-based strategies for NPC patients.

In 1889, a “seed and soil” hypothesis was proposed by Paget, suggesting that microenvironment were important for tumor development [25]. Tumor microenvironment contains various non-malignant cell types, including endothelial cells, macrophages, fibroblasts, adipose tissue, and mesenchymal stem cells [26]. Among them, fibroblasts, endothelial cells and macrophages are the most well-recognized cell types interacting with tumor cells by SEVs signaling [26, 27]. The ultimately results of these cell-by-cell interactions are decided by the origin of these SEVs and SEVs cargo [28].

Based on the above theory, SEVs miRNA act as key regulators in NPC metastasis, and SEVs miRNA may be taken up by non-malignant cell to prepare pre-metastatic niche. However, are there any miRNA specific dysregulated in NPC-SEVs rather than free in serum? Does SEVs miRNA uptake by non-malignant cell synchronously or not? In this study, we aimed to identify specific SEVs miRNA for NPC patients, and explain the underlying mechanisms for functional accumulation of these miRNAs in SEVs.

## Results

### MiR-6750 is specific down-regulated in NPC-SEVs and inhibits NPC metastasis

**Fig. 1:**
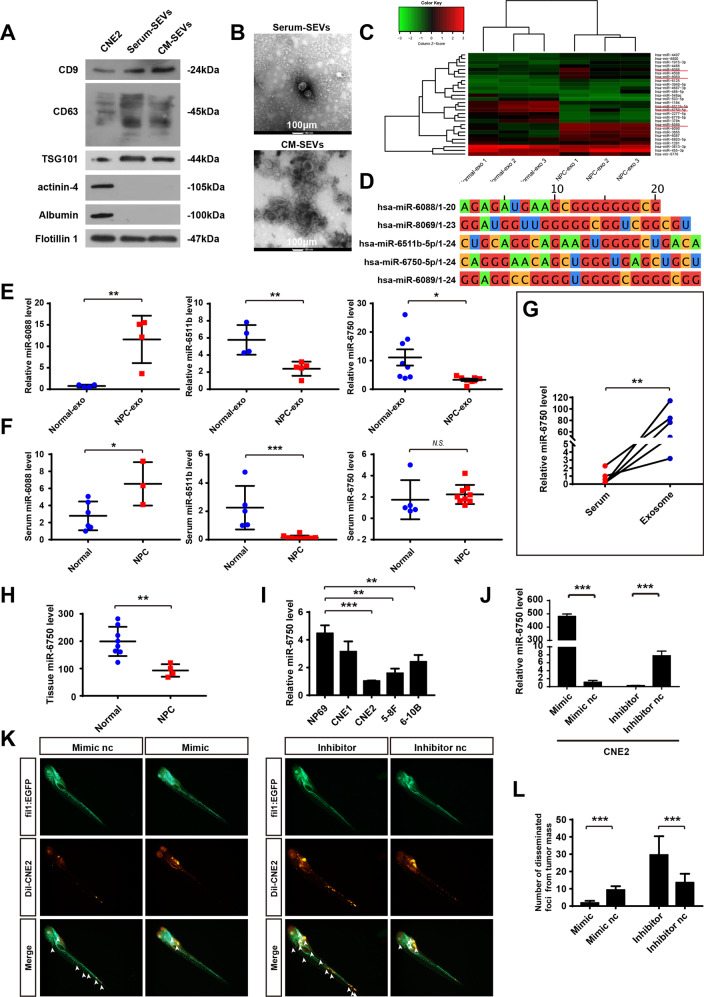
MiR-6750 is specific down-regulated in NPC-SEVs and inhibits NPC metastasis. **A** Western blot analysis of CD63, CD9, TSG101, Albumin, actinin-4 and Flotillin 1 in CNE2 cell line and SEVs. **B** Representative electron microscopy image of SEVs. **C** genome-wide analysis of miRNA expression in NPC-SEVs vs. healthy volunteers. **D** The sequences of miR-6088, miR-8069, miR-6511b, miR-6750 and miR-6089. **E**, **F** Levels of miR-6088/6511b/6750 in serum-SEVs and serum were measured by qRT-PCR. **G** Levels of miR-6750 in serum-SEVs vs. serum. **H**, **I** Levels of miR-6750 in NPC tissues and cells. **J** Transfection efficiency was measured by qRT-PCR. **K** Gross-observation tumor cell dissemination and metastasis. CNE2 cells with different miR-6750 level labeled with Dil were injected into zebrafish embryos. 2 days later, migration of CNE2 cells was measured by fluorescence microscopy. Arrowheads indicate disseminated tumor foci. **L** Quantitative evaluation of NPC cell dissemination and metastasis. Student’s t-test, mean ± SEM.

To identify specific altered SEVs miRNAs in NPC and conduct potential non-invasive diagnostic and therapeutic tool, we isolated and characterized SEVs by marker expression and electron microscopy (Fig. 1A, B). After performing Affymetrix miRNA Microarrays using three pairs of circulating SEVs from NPC patients and healthy volunteers, we found 18 up-regulated miRNAs and 10 down-regulated miRNAs in NPC-SEVs vs. healthy volunteers (Fig. 1C). Among them, miR-6088, miR-8069, miR-6511b, miR-6750, and miR-6089 stood out for biological function unknown (Fig. 1D). Firstly, to determine the expression of five miRNAs, primes were designed. Among them, primers of miR-6089 and miR-8069 were designed failure for containing too many C and G bases. MiR-6088 and miR-6511b expression level in serum and serum-SEVs was consistent, indicating miR-6088 and miR-6511b in serum do reflect that in serum-SEVs, and examining circulating miR-6088 or 6511b level may be more convenient (Fig. 1E, F). Notably, qRT-PCR assay showed miR-6750 specifically down-regulated in NPC-SEVs but not in serum (Fig. 1E, F), and miR-6750 level in SEVs was significantly higher than that free in serum (Fig. 1G). So, we chose miR-6750 for further study.

Then, to investigate the role of miR-6750-SEVs in NPC progression, we analyzed the expression level and biological function of miR-6750. QRT-PCR assay validated miR-6750 was attenuated in NPC tissues and cell lines (Fig. 1H–I). And CNE-2 was chosen to transfected with miR-6750 mimic, negative control (nc), and miR-6750 inhibitor to obtain cells with different levels of miR-6750 for the lowest miR-6750 level (Fig. 1J). After injecting cells in a zebrafish model, we found that miR-6750 inhibited NPC metastases through monitoring cell dissemination, invasion, and metastasis at the single cell level (Fig. 1K–L).

Collectively, we knew miR-6750 was significantly dysregulated in NPC cells, miR-6750 attenuated NPC metastasis and miR-6750 was significantly enrichment in NPC-SEVs vs. free in serum.

### MiR-6750-SEVs can be shuttled to HUVECs in TME to regulate angiogenesis

**Fig. 2:**
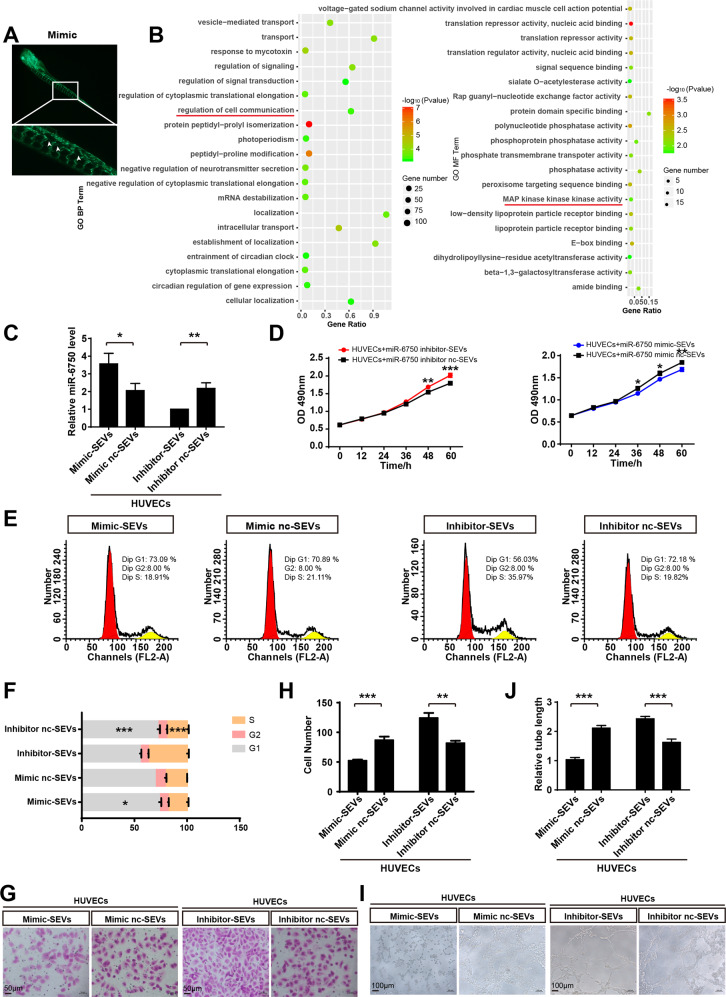
MiR-6750-SEVs can be shuttled to HUVECs in TME to regulate angiogenesis. **A** CNE2 with higher miR-6750 level inhibit angiogenesis in zebrafish. **B** GO annotation analysis for miR-6750 target genes in the biological process and molecular function. **C** After co-cultured with CM-SEVs from CNE2 cells, miR-6750 levels in HUVECs were measured by qRT-PCR. Student’s t-test. **D** CCK8 assay was performed to measure viabilities of HUVECs treated as graph-depicted groups. Two-way ANOVA. **E**, **F** Cell-cycle analysis was used. One-way ANOVA. **G**, **H** Transwell migration assays were performed to measure cell migration. Student’s t-test. **I**, **J** Tube formation assays were performed using Matrigel. Student’s t-test.

To explore the underlying mechanism of miR-6750 in NPC metastasis, we enlarged zebrafish image, indicating CNE2 with higher miR-6750 level inhibit angiogenesis in Tg (fli1a: EGFP) transgenic zebrafish (Fig. 2A). Biological process (BP) analysis and molecular function (MF) analysis showed miR-6750 target genes were enriched in regulation of cell communication, and MAP kinase kinase kinase activity, which also supported our conjecture that miR-6750 modulate angiogenesis (Fig. 2B). As the MAPK pathway includes three main kinases, MAPK kinase kinase, MAPK kinase and MAPK, and the ERK-MAPK/P38-MAPK pathway were confirmed as important signaling cascades that plays crucial roles in angiogenesis/ macrophage polarization [29, 30]. Then, we give a conjecture that the physiological purpose of generating SEVs accumulating miR-6750 can regulate both angiogenesis and macrophage polarization to inhibit NPC metastasis.

Angiogenesis is the outgrowth of new blood vessels branching off from pre-existing ones or deriving from endothelial progenitor cells. In this way, HUVECs offer oxygen, nutrients, or cell metastatic conduits for NPC metastasis [31, 32]. To explore the potential effects of miR-6750-SEVs on angiogenesis, SEVs containing miR-6750 extracted from CNE2-CM were added to HUVECs. QRT-PCR assay showed a relatively high miR-6750 level was observed for mimic-SEVs treatment group vs. inhibitor-SEVs group (Fig. 2C). In vitro cellular analyses showed that high miR-6750-SEVs attenuated cell growth, migration, and tube formation of ECs, while silencing miR-6750 expression enhanced these functions (Fig. 2D–J), which was in agreement with the role of miR-6750 in angiogenesis (Fig. S1).

### MiR-6750-SEVs can be shuttled to Macrophage in TME to regulate macrophage phenotype

**Fig. 3:**
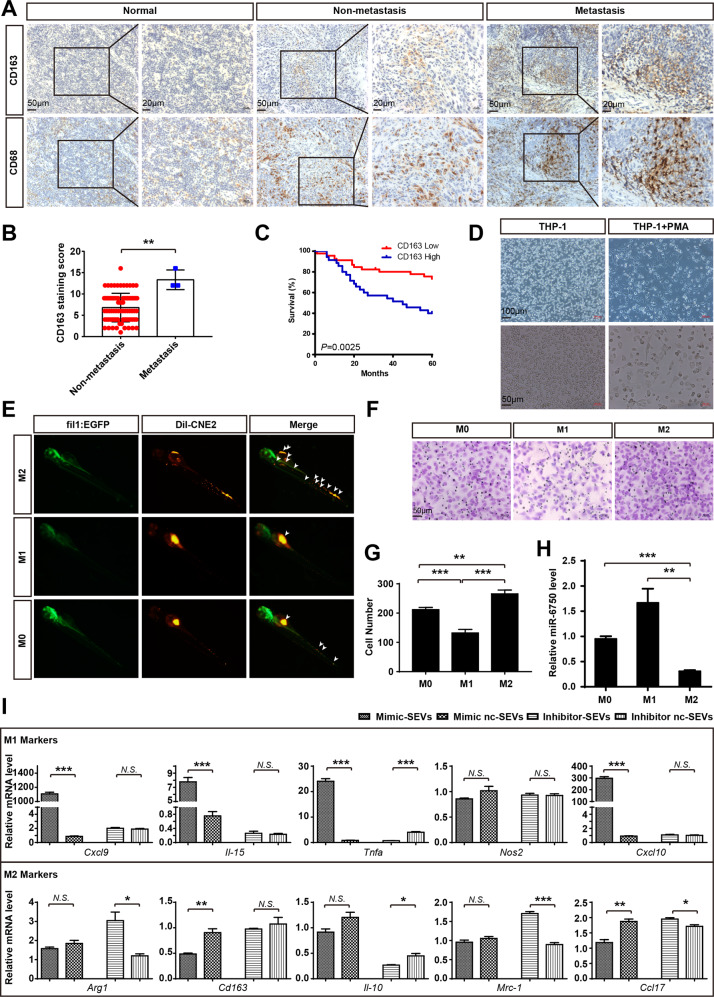
MiR-6750-SEVs can be shuttled to Macrophage in TME to regulate macrophage phenotype. **A** Representative images of CD163/CD68 in tissues collected from graph-depicted groups. **B** Student’s t-test between CD163 expression and NPC metastasis. **C** Statistical analyses of the association of CD163 level with survival time of the patients. Log-rank test. Staining score of CD163 in NPC tissue was defined as low expression (scores of 0–8) or high expression (scores of 9–16) by the X-tile Software. **D** Representative image of macrophages was shown (THP-1 cells were treated with or without PMA). **E** NPC cell dissemination and metastasis was analyzed by a zebrafish model. Arrowheads indicate disseminated tumor foci. **F**, **G** Transwell migration assays were performed to measure cell migration. Student’s t-test. **H** miR-6750 levels in Macrophage were measured by qRT-PCR. Student’s t-test. **I** M0 Macrophages treated with various SEVS was used to measure Macrophage phenotype by qRT-PCR assay. Student’s t-test.

As the prominently abundant immune cell in TME, macrophages can be classified into M1 phenotype (classically activated) or M2 (alternatively activated) phenotype [33]. M2 phenotype is widely accepted to exhibit tumour-promoting characteristics, such as tumour proliferation, invasion and metastasis [34, 35]. Firstly, to know the distribution of M2 macrophage, we investigate the expression of its representative marker (CD163), showing elevated CD163 overexpression in NPC specimens, especially higher in metastatic NPC tissues (Fig. 3A). Moreover, we evaluated the relationship between CD163 expression and clinicopathologic variables in NPC. Results confirmed high CD163 expression was significantly associated with distant metastasis (*P* = 0.04) (Fig. 3B), and clinical stage (*P* = 0.0072) (Fig. S2A). Further analysis showed that patients with high CD163 infiltration exhibited poor overall survival time (*P* = 0.0025, Fig. 3C), which was consistent with Haoran Huang’s study. These clinical findings indicate that M2 macrophage might regulate NPC metastasis. To verify this opinion, human THP-1 monocytes was treated with PMA to generate M0 macrophages, which were then co-cultured with LPS or IL-4 to generate M1 macrophages or M2 macrophages (Fig. 3D and Fig. S2B). In vitro and in vivo analyses further confirmed that M2 macrophage enhanced proliferation, invasion and metastases in NPC cell (Fig. S2C and Fig. 3E–G).

A plethora of evidence indicates that macrophages within microenvironment are activated and polarized by signals from TME [33, 36, 37]. To evaluate the effect of miR-6750-SEVs on macrophage polarization, M0 macrophage was co-culture with SEVs extracted from CNE2 expressing different miR-6750 level. QRT-PCR assay showed that macrophage with miR-6750 mimic-SEVs exhibited higher M1 marker gene expression (CXCL9, IL-15, TNFa, CXCL10), and lower M2 marker gene expression (CD163, CCL17) (Fig. 3H). Thus, these results demonstrated that macrophage co-cultured with mimic-SEVs were skewed toward to M1 phenotype, which happen to be consistent with higher miR-6750 in M1 macrophage examined by qRT-PCR (Fig. 3I).

Above all, the data showed that enhanced miR-6750 level in NPC-SEVs inhibits NPC metastasis; miR-6750-SEVs could be shuttled to HUVECs or macrophage to inhibit pre-metastatic niche. Then, how SEVs were delivered to HUVECs and macrophage, equally or one cell had higher priority.

### MiR-6750-SEVs-incorporating macrophages inhibit angiogenesis

**Fig. 4:**
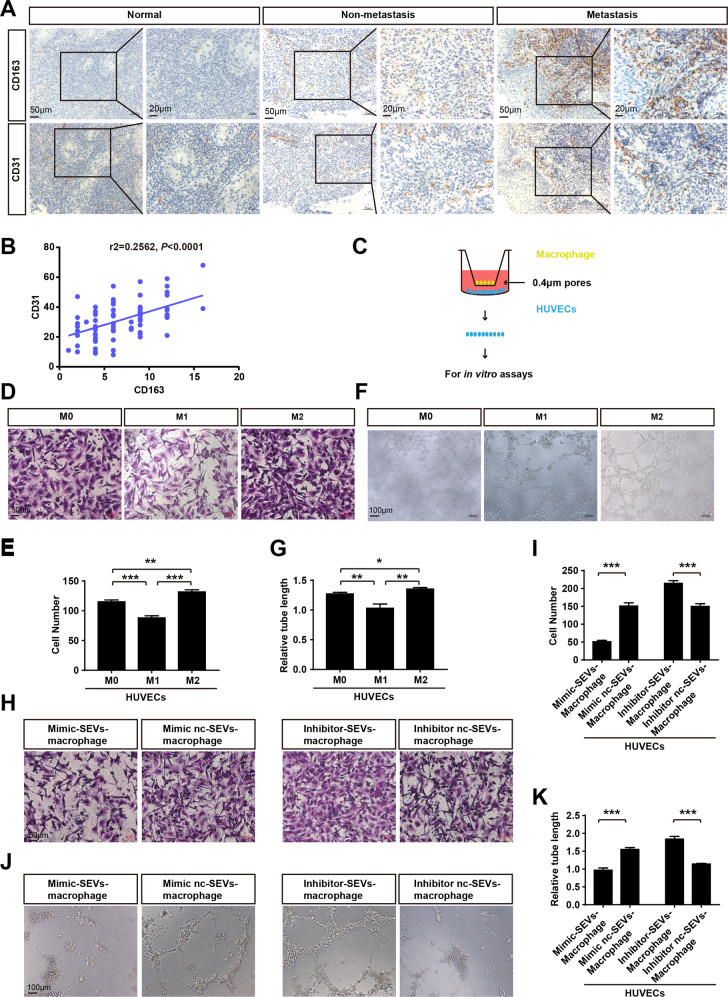
MiR-6750-SEVs-incorporating macrophages inhibit angiogenesis. **A** Representative images of CD163/CD34 in tissues collected from graph-depicted groups. **B** Pearson correlation between miR-CD163 expression and MVD. Linear regression. **C** Scheme of HUVEC collection after co-cultured with macrophage. **D**, **E** Transwell migration assays were performed to measure cell migration. Student’s t-test. **F**, **G** Tube formation assays were performed using Matrigel. Student’s t-test. **H**, **I** Transwell migration assays were performed to measure cell migration. Student’s t-test. **J**, **K** Tube formation assays were performed using Matrigel. Student’s t-test.

There is convincing evidence that macrophages stimulate tumor initiation and malignant progression. As tumors progress to malignancy, HUVECs could be regulated by the presence of macrophages for two roles: promoting pathological angiogenesis and enhancing tumor cell extravasation, colonization [38–41]. To explore the underlying association between HUVECs and macrophages in NPC, Pearson correlation analysis was performed, showing higher M2 macrophage infiltration exhibits higher microvessel density (MVD) (Fig. 4A–B). In vitro assays showed that M2 macrophage enhanced cell migration and tube formation of HUVECs, while M1 macrophage suppressed these functions (Fig. 4C–G). What’s more, cellular analyses showed that high miR-6750-SEVs -incorporating macrophages attenuated cell migration and tube formation of ECs, while low miR-6750-SEVs -incorporating macrophages enhanced these functions (Fig. 4H–K). On the basis of this, macrophage may mediate uptake of miR-6750-SEVs by HUVECs, which partly explain the function of macrophage promoting angiogenesis.

### Macrophage can transmit SEVs to HUVECs

**Fig. 5:**
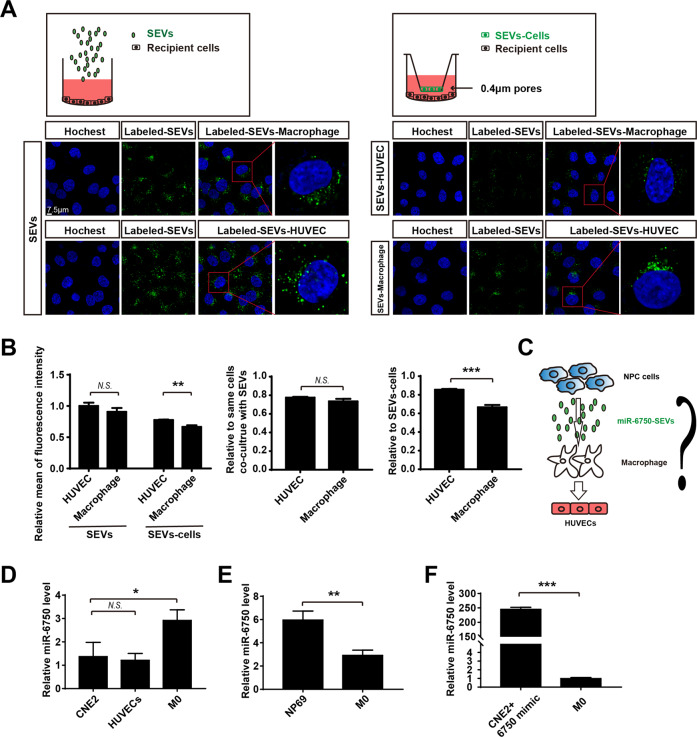
Macrophage can transmit SEVs to HUVECs. **A** Uptake of SEVs in graph-depicted groups by confocal microscopy. Blue: Hoechst staining; green: PKH67-labeled SEVs. **B** Statistical analyses of uptake efficiency. Student’s t-test. **C** Scheme of miR-6750-SEVs transmission (NPC cells-Macrophage-HUVECs). **D** Levels of miR-6750 in CNE2, HUVECs and M0 macrophage. **E** Levels of miR-6750 in NP69 vs. M0. **F** Levels of miR-6750 in CNE2 transfected with miR-6750 mimic vs. M0.

In order to verify our hypothesis, we isolated SEVs present in CM (conditioned medium) from CNE2 cells to establish different co-culture models as showed in Fig. 5A: PKH-67-labeled SEVs, SEVs-incorporating HUVECs (SEVs-HUVEC) and SEVs-incorporating macrophages (SEVs-Macrophage). To ascertain the transport of SEVs in TME, we examined fluorescence intensity, which monitored SEVs uptake. Results showed the uptake efficiency of PKH-67-labeled SEVs was no statistical differences between HUVECs and macrophage, while HUVECs co-culture with SEVs-Macrophage exhibited higher uptake efficiency than macrophage coculture with SEVs-HUVEC (Fig. 5A, B). This result suggested that macrophage mediate SEVs contain miR-6750 transmit to HUVECs in TME and the function of macrophage promoting metastasis maybe partly altered to SEVs transport to HUVECs (Fig. 5C). Then, to further verify the SEVs-mediated miR-6750 transfer in TME, we analyzed the endogenous expression level of miR-6750 in CNE, HUVECs and M0 macrophage, showing that miR-6750 level was higher in macrophage than CNE2 or HUVECs and with no significant difference between CNE2 and HUVECs (Fig. 5D). Only making miR-6750 level close to or higher than normal nasopharyngeal epithelial cells (NP69) by addition of exogenous miR-6750 level, miR-6750-SEVs were transferred from NPC cells to macrophage and then to HUVECs happened (Fig. 5E–F).

**Fig. 6:**
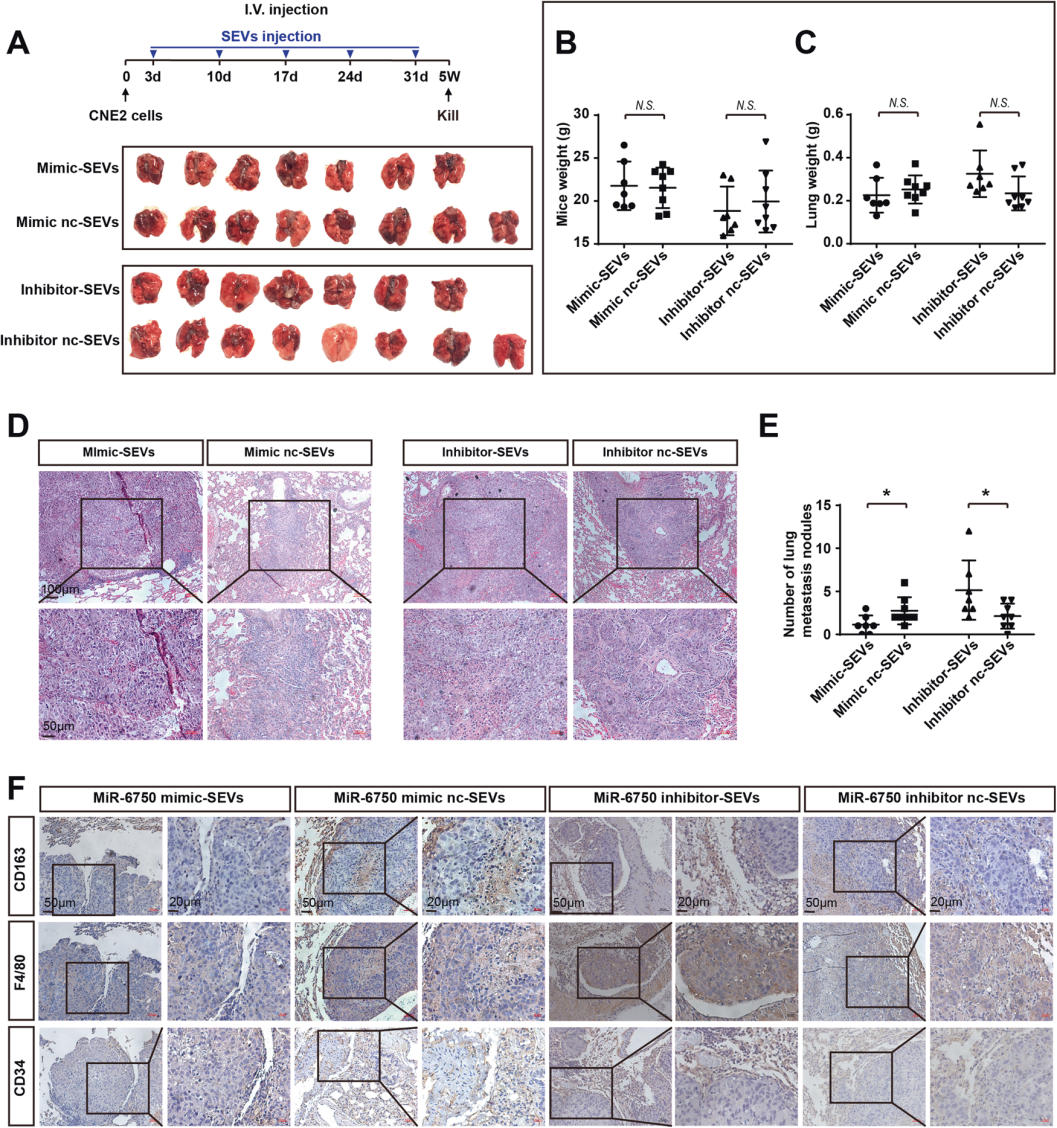
MiR-6750-SEVs inhibits NPC metastasis by regulating angiogenesis and M2 macrophage infiltration. **A** Schema of animal experiment. BALB/c nude mice receiving 20 μg of purified SEVs isolated from CNE2-CM weekly 3 day after CNE2 injection. And gross-observation lung metastasis was showed in graph-depicted groups. **B**, **C** Statistical analyses of mice weight and lung weight. Student’s t-test. **D** Representative images of HE staining. **E** Statistical analyses of Number of lung metastasis nodules in graph-depicted groups. Student’s t-test. **F** Representative images of CD163, F4/80 and CD34 in tissues collected from graph-depicted groups.

Next, to further demonstrate the relationship among miR-6750-SEVs, HUVECs, Macrophage and NPC metastasis, we tail injected CNE2 cells and SEVs with different miR-6750 level into BALB/c mice models as shown in Fig. 6A. 5 weeks later, we found that mice injected with miR-6750-overexpressing SEVs dramatically decreased number of lung metastasis nodules, whereas mice weight and lung weight showing no difference (Fig. 6B–E), indicating that miR-6750-SEVs inhibited NPC metastasis in vivo. Additional IHC assay revealed that there were higher M2 macrophage infiltration and higher density of MVD in mice with low miR-6750 SEVs vs. high miR-6750 SEVs (Fig. 6F), in turn, supports our conjecture that miR-6750-SEVs regulate NPC metastasis through the establishment of anti-metastatic niche.

### M6PR is the direct target of miR-6750

**Fig. 7:**
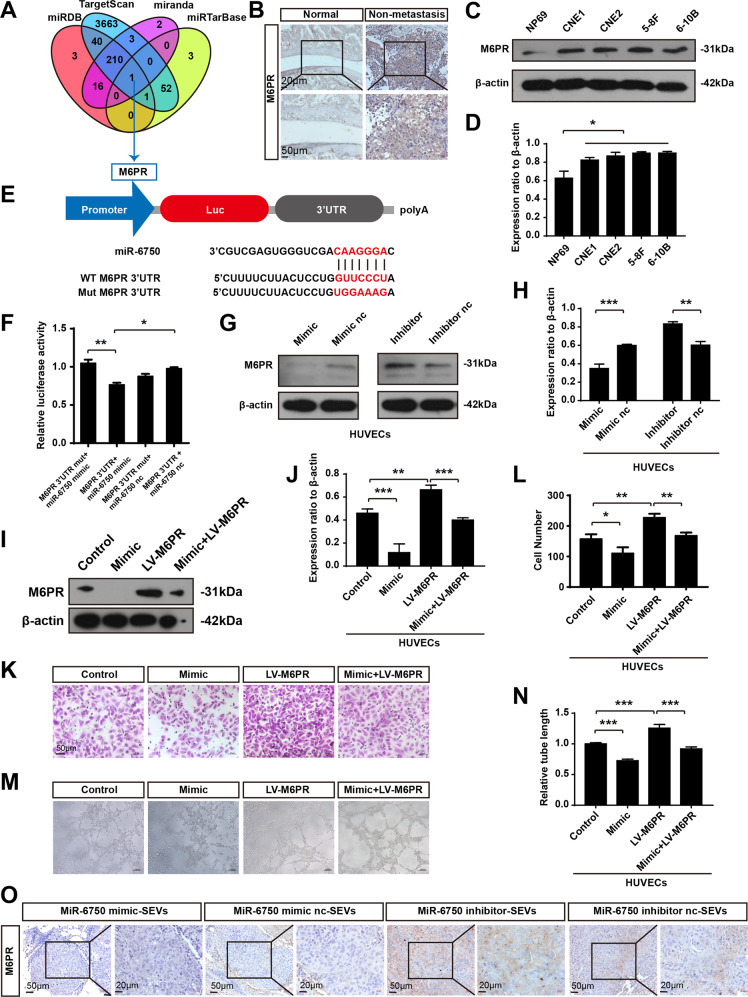
MiR-6750 directly targets M6PR. **A** Venn diagram depicting showing predicted MIR-6750 targets. **B** Representative IHC images of M6PR in NPC tissues. **C**, **D** Levels of M6PR in NPC cells measured by western blot. Student’s t-test. **E**, **F** Overexpression of miR-6750 reduced M6PR-3′-UTR luciferase activity in vitro but not mutated M6PR-3′-UTR luciferase activity. Student’s t-test. **G**, **H** M6PR immunoblotting in HUVECs treated as indicated was analyzed. Student’s t-test. **I**, **J** M6PR immunoblotting in HUVECs treated as indicated was analyzed. One-way ANOVA. **K**, **L** Transwell migration assays were performed to measure cell migration. Student’s t-test. **M**, **N** Tube formation assays were performed using Matrigel. One-way ANOVA. **O** Representative IHC images of M6PR in mice metastatic lung tissues.

MiRNA exerts a majority of biological processes via post-transcriptional repression of target gene expression. To identify the direct targets of miR-6750, four common bioinformatics tools (i.e., MiRDB, Targetscan, miranda, and miRTarBase) were used, and mannose 6-phosphate receptor (M6PR) was the only one selected (Fig. 7A). Mannose 6-phosphate receptor (M6PR) includes cation-dependent mannose-6-phosphate receptor (CD-M6PR) and cation-independent mannose-6-phosphate receptor (CI-M6PR). Generally, M6PR is considered as CD-M6PR, which is reported to mediate cellular factors transporting from the trans-Golgi network (TGN) to late endosomes (LEs) [42]. And the function of M6PR in tumor metastasis and TME is largely unclear. To know the role of M6PR in NPC progression and the potential relationship between miR-6750 and M6PR, firstly, the expression level of M6PR in NPC was analyzed. IHC provided evidence that M6PR was enriched in NPC samples, which was contrary to miR-6750 level (Fig. 7B). Then, western blot analysis was applied to validate M6PR level, showing that M6PR was enriched in NPC cell lines as well (Fig. 7C–D). Luciferase reporter assays showed enhanced biologically effective interaction between miR-6750 and M6PR-3’-UTR (Fig. 7E–F). M6PR protein levels was also dramatically inhibited in cells with high miR-6750 level, whereas M6PR increased in cells with low miR-6750 level (Fig. 7G–H). Moreover, we analyzed the effect of miR-6750 on M1 polarization, showing miR-6570 activated macrophage toward to M1 phenotype, which could be abolished by LV-M6PR treatment, indicating that miR-6750 exerted its function by directly targeting M6PR (Fig. S3). And, transwell and tube formation assays showed that up expression of M6PR by LV-M6PR could restore the angiogenesis blocked by miR-6750 mimic (Fig. 7I–N). IHC assay revealed that there were higher M6PR level in mice with low miR-6750 SEVs vs. high miR-6750 SEVs (Fig. 7O). All the data suggest that M6PR is the direct target of miR-6750. As MF analysis in Fig. 2 shows that miR-6750 target genes were enriched MAP kinase activity, we then undertook to determine whether M6PR regulate MAPK (ERK, JNK and p38) pathway. We discovered that miR-6570 mainly alleviate ERK signaling, which could be abolished by LV-M6PR treatment (Fig. S4A–D).

In addition, we also analyzed CI-M6PR level in NPC for involving the manipulation of intracellular protein degradation for tumour therapeutics [43]. We explored the expression level of CI-M6PR, showing CI-M6PR was also enriched in NPC samples and NPC cells (Fig. S5A–C). Furthermore, CI-M6PR protein levels was also inhibited in cells with high miR-6750 level (Fig. S5D–E). Moreover, IHC assay revealed that there were higher CI-M6PR level in mice with low miR-6750 SEVs vs. high miR-6750 SEVs (Fig. S5F). These data suggest that CI-M6PR may also be target for miR-6750. However, we discovered that the potentially binding sites of CI-M6PR-3‘-UTR is poorly specific (Fig. S5G), and CI-M6PR was not chosen for the follow-up study.

### The miR-6750-M6PR axis suppresses metastasis by remodeling TME

**Fig. 8:**
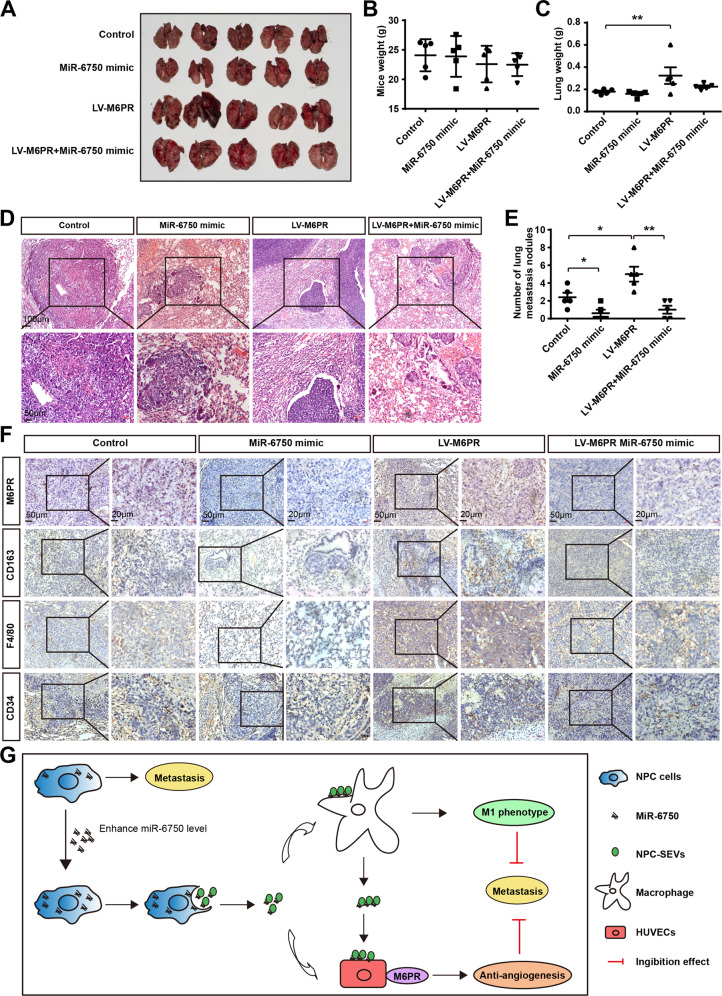
MiR-6750 directly targets M6PR to regulate NPC metastasis. **A** Gross-observation lung metastasis in graph-depicted groups. **B**, **C** Statistical analyses of mice weight and lung weight. Student’s t-test. **D** Representative images of HE staining. **E** Statistical analyses of Number of lung metastasis nodules in graph-depicted groups. Student’s t-test. **F** Representative images of M6PR, CD163, F4/80, and CD34 in tissues collected from graph-depicted groups. **G** Schematic diagram of the mechanism for miR-6750-M6PR axis inhibiting metastasis by establishing an anti-metastasis microenvironment.

Subsequently, to further explore the potential role of M6PR in miR-6750-mediated metastasis, we injected CNE2 cells transfected with control miRNA, miR-6750 mimic, LV-M6PR and LV-M6PR + miR-6750 mimic, respectively, into the tail of BALB/c mice. After 5 weeks later, we demonstrated that miR-6750 mimic reduced number of lung metastasis nodules rather than mice weight or lung weight. Interestingly, M6PR overexpression significantly rescued cell metastasis, showing that miR-6750 regulated metastasis by directly targeting M6PR (Fig. 8A–E). Analogously, lower M2 macrophage infiltration and lower density of MVD the migration in mice with miR-6750 mimic treated could be reversed by M6PR treatment (Fig. 8F). All these results indicated that miR-6750-M6PR axis can mediated NPC metastasis by remodeling TME (Fig. 8G).

## Discussion

Accumulating evidence indicates that SEVs miRNAs exert an important role in tumor progression [16, 44–46]. Oncogenic miRNAs, or tumor suppressor miRNAs can be transferred by SEVs to neighboring or distant recipient cells and functioned in them. Interestingly, we have isolated, identified NPC-exo before, and proved that oncogenic miR-23a can be shuttled to HUVECs to promote metastasis-associated angiogenesis through exosomes [24]. To further identify specific altered SEVs miRNAs in NPC and conduct potential non-invasive diagnostic and therapeutic tools, we performed genome-wide analysis of miRNA expression in this study using three pairs of circulating SEVs from NPC patients and healthy volunteers. Among them, 18 up-regulated miRNAs and 10 down-regulated miRNAs in NPC-SEVs vs. normal-SEVs. Interestingly, miR-6088, miR-8069, miR-6511b, miR-6750, and miR-6089 stood out for biological function unknown. After comprehensively analyzing the five miRNAs in serum and circulating SEVs, we picked miR-6750 that was showing differential pattern in NPC-SEVs, enrichment in NPC-SEVs but not in serum and biological function largely unknown (Fig. 1).

Advances in in vitro and in vivo analyses have results in the generation of different genes that function in different tumour progression, such as proliferation, apoptosis, autophagy, and metastasis [47–50]. As distant metastasis is the main reason for NPC treatment failure, gene investigation can construct diagnostic indicators and provide individualized treatment methods [5]. We tried to explore the likelihood effects of miR-6750 on NPC metastasis. It was noteworthy that miR-6750 inhibited NPC metastases through monitoring CNE2 cell dissemination, invasion, and metastasis at the single cell level using a zebrafish model (Fig. 1). At this point, our results firstly indicated that miR-6750 was significantly downregulated in NPC-SEVs and miR-6750-SEVs suppressed NPC metastasis. Then, we gone on to clarify underlying mechanisms for miR-6750-SEVs-mediate metastasis. Considering that SEVs have been proven to exert an important role in remodeling the tumor microenvironment and miR-6750 was higher gathered in SEVs rather than serum level, miR-6750-SEVs may be shuttled to distant cells.

During cancer progression, metastasis of cancer cells may attribute to co-evolution of tumor cells and their direct environment. TME comprises different structures surrounded, including blood vessels, immune cells, fibroblasts, extracellular matrix (ECM), and signaling molecules. Among them, convincing evidence showed high MVD and M2 macrophage infiltration was observed in advanced NPC patients [51, 52]. And we examined the effect of miR-6750-SEVs on angiogenesis and macrophage polarization. First, endothelial cells (ECs), one important cell type in TEM, is the necessary components of tumor angiogenic vessels. In this way, ECs offers nutritional support and conduits for tumor growth and metastasis. Based on CCK8, transwell and tube formation assays, we confirmed that miR-6750-SEVs inhibits angiogenesis (Fig. 2). The second is immune cells, headed by macrophage. In accordance with previous studies [52], we also demonstrated M2 biomarker CD163 was elevated in NPC specimens and patients with high M2 macrophage infiltration exhibited poor prognosis. Moreover, our unique finding is that miR-6750-SEVs attenuate macrophage polarization to M2 (Fig. 3).

As described above, we found low level of miR-6750 in NPC-SEVS inhibits NPC metastasis; miR-6750-SEVs could be shuttled to HUVECs or macrophage to inhibit pre-metastatic niche. Then, how does SEVs transport, equally uptake by these two cells or one cell had higher priority. Evidence from previous studies showed that macrophage have diverse ways to regulate tumor metastasis, directly suppress anti-tumor immune mechanisms to promote cell escape and indirectly regulate stromal cells proliferation to destroy normal tissue physiological homeostasis [53–56]. Once the macrophage received factors released by tumor cells, the macrophage can be recruited to promote angiogenesis [57, 58]. After analyzing the relationship between macrophage and HUVECs in NPC, we found higher M2 macrophage infiltration exhibits higher MVD in NPC and M2 macrophage enhanced cell migration and tube formation of HUVECs (Fig. 4). After analyze the uptake efficiency of recipient cells with different treatment, we found HUVECs coculture with SEVs-macrophage exhibited higher uptake efficiency than macrophage coculture with SEVs-HUVEC, showing miR-6750-SEVs in TME can be transport to HUVECs by macrophage. (Figs. 5 and 6).

There may be additional target genes regulated by miR-6750 assist its anti-metastatic effect. Bioinformatics analysis by four common tools (i.e., MiRDB, Targetscan, miranda, and miRTarBase) indicated one common target gene M6PR. The function of M6PR in NPC metastasis and TME is unclear before. We identified for the first time that miR-6750 could directly bind to the specific site in M6PR-3′-UTR. After examining M6PR protein level, we showed M6PR was enriched in NPC samples, which was inverse to miR-6750 expression level (Fig. 7). Further analysis showed that M6PR were significantly downregulated in miR-6750 mimic-transfected cells and M6PR could restore the angiogenesis blocked by miR-6750 mimic in vitro or anti-metastasis ability in nude mice (Fig. 8). Therefore, our study indicated that M6PR remodels TME to promote NPC metastasis, and the anti-metastasis functions of miR-6750 can be ascribed to direct repression of M6PR. Recently, researchers attempt to use SEVs miRNAs for cancer therapy. Either Oncogenic or tumor suppressor miRNAs can be transferred to receipted cells by SEVs and function in them. Then, destroying miR-6750-M6PR axis may be a promising role to suppress NPC metastasis.

Collectively, this study revealed miR-6750 was specific dysregulated in NPC-SEVs but not in serum, and miR-6750-SEVs can remodel TME to modulate NPC metastasis by directing targeting M6PR. Our study provides a therapeutic possibility of destroying miR-6750-M6PR axis in the TME; these leads, in turn, to provide a new selective mean for developing TME‐based therapeutics to prevent and block NPC metastasis.

## Materials and methods

### Tissue specimens

For expression analysis, we collected human tissue samples and serum samples from Affiliated Hospital of Nantong University, China. Among them, paraffin-embedded biopsy tissues were diagnosed between Aug 3, 2005, and Dec 17, 2010. All patients included had not received any anti-tumour therapy prior to biopsy sampling.

### Cell lines and cell culture

Four NPC cell lines (CNE1: high differentiation, CNE2: low differentiation, 5–8 F: high tumorigenesis and high metastasis, and 6–10B: low tumorigenesis and low metastasis) and the immortalized normal nasopharynx epithelial NP69 cells used in this study were cultured in Department of Otolaryngology Laboratory, Affiliated Hospital of Nantong University. For culturing, NPC cell lines were maintained in RPMI 1640 (BI Biological Industries) supplemented with 10% FBS (Gibco) or 10% exosome-free serum (SBI SystemBiosciences). NP69 cells were maintained in keratinocyte-SFM (Thermo Fisher Scientific,17005-042). CNE2 cell line had been authenticated by cellular morphology and the short tandem repeat (STR) analysis. In addition, HUVECs were maintained in EC medium (ScienCell Research Laboratories). THP-1 cells obtained from Shanghai Zhong Qiao Xin Zhou Biotechnology Co.,Ltd (ZQ0086) were cultured in RPMI-1640 medium (ZQ-206) containing 10%FBS, 100 U/mL penicillin, 100 μg/ml streptomycin and 50 μmol/L β-Mer.

### Macrophage differentiation and polarization conditions

THP-1 treated with 100 ng/mL phorbol-12-myristate-13-acetate (PMA) overnight was defined as M0 macrophages. To induce macrophage polarization, M0 macrophages were treated with 50 ng/mL LPS (Sigma) plus 20 ng/mL IFN-γ (Peprotech) or 20 ng/mL IL-4 (Peprotecch) to achieve M1 or M2-polarized macrophages, respectively. After 18 h, polarized macrophages were harvested for further analysis.

### Immunohistochemistry (IHC)

IHC staining was performed as reported previously [59] using a 1:500 antibody dilution for CD34 (Abcam), 1:100 dilution for CD68 (Abcam), 1:100 dilution for F4/80 (Proteintech), 1:100 dilution for M6PR (ABclonal) and 1:500 dilution for CI-M6PR (Abcam). Staining intensity and abundance of positive cells were used to assess stained slides. The staining intensity was categorized as 4 (strong), 3(moderate), 2 (weak), or 1 (negative), while positive cell proportion use the following scale: 1 (0–25%), 2 (26–50%), 3 (51–75%), and 4 (76–100%) by two pathologists blind to the patient’s clinicopathological information. A final score was derived from intensity score multiplied by the percentage score.

### Transfection and transduction with plasmids, miRNA inhibitors, and mimics

All transfection experiments were carried out as done previously [24]. MiR-6750 mimic/nc/inhibitor were designed by Biomics Biotechnologies (Nantong, China). LV-M6PR supplied from Genechem (Shanghai, China) was used for overexpressing M6PR level.

### Western blot analysis

Western blot analysis was performed as previously described [60] using a 1:1000 antibody dilution for M6PR (ABclonal), 1:1000 antibody dilution for CI-M6PR (Abcam), 1:1000 dilution for β-actin (Santa Cruz Biotechnology). To analyze relative protein level, ImageJ software was applied.

### Quantitative real-time PCR (qRT-PCR)

QRT-PCR assay was constructed as previously described [24, 47]. Briefly, total RNA in different cells was isolated with TRIzol reagent (Invitrogen), circulating miRNA was extracted using the the miRcute Serum/Plasma miRNA isolation Kit (TIANGEN, DP501), and SEVs miRNAs were isolated by exosomal miRNA and Total Exosome RNA Kit (Ambion) and MirVana RNA isolation kit (Ambion). Primers for miRNAs were designed by Biomics Biotechnologies, whereas primers for macrophage polarization was purchased from Guangzhou RiboBio. Cellular RNA level were normalized to RNU6, while circulating and SEVs miRNAs were normalized to cel-miR-39 (synthetic spike control).

### Microvasculature density counting, CCK8 assay, transwell assay, cell cycle analysis, and tube formation assay

All assays were done as described previously [24]. For CCK8 assay, 2,000 cells were seeded and incubated for 24 h in 96-well. A total of 10 μl CCK-8 Kit reagents (Biosharp, Guangzhou, China) were added and incubated for 2 h. Then, the absorbance was read at 450 nm.

For migration transwell, HUVECs was washed and resuspended in medium with no serum. A total of 5 × 10^4^ cells was seeded in the upper chambers with a pore size of 8 µm. Medium supplying 10% FBS with or without exosomes was added in the lower chambers. 16 h later, we removed cells on the upper side, fixed and stained cells on the underside of chamber. Five random fields were counted to analyze cell migration.

For endothelial cell tube formation assay, HUVECs treated with miR-6750 mimic/inhibitor or nothing were harvested, counted, resuspended and seeded (2 × 10^4^ cells/well) on Matrigel in 96-well plates. Once HUVECs attached, cellular medium was removed, and ECM with or without SEVs would be supplied. Inverted microscope was used to observe and analyze tube-like structures.

### EV separation, quantitation, and characterization

EVs separation, quantitation and characterization followed the MISEV2018 guidelines [8]. And trying to align with MISEV guidelines and suggests, we used the term small extracellular vesicles in this study instead of exosomes due to the heterogeneity of vesicles obtained after existing purification methods.

SEVs were mainly isolated by ultracentrifugation method. For SEV separation, NPC Cells were cultured and grown to an approximate 90% confluence, then removed and replaced by RPMI 1640 supplemented with exosome-free serum. After incubation for 48 h, the supernatant was collected. The supernatant or serum was centrifuged at 4 °C at 300 g for 5 min, 3000 g for 20 min, 6000 g for 40 min and then 10,000 × *g* for 60 min. Then, the supernatant was ultracentrifuged at 100,000 × *g* 4 °C for 60 min. The precipitation was washed twice with 1 ml PBS and ultracentrifuged at 100,000 × *g* 4 °C for 60 min. The NPC-SEVs pellets were resuspended in PBS and stored at −80 °C. SEVs were also isolated by size-exclusion chromatography column following the manufacturer’s directions as shown in our previous study [24] to confirm the effects of SEV that isolated with ultracentrifugation method.

NPC-EVs characterization and quantitation were determined by transmission electron microscope analysis and western blotting analysis as shown in our previous study [24, 61].

### SEV labeling and Immunofluorescence (IF)

To explore SEVs transport in TME, SEVs isolated from CM were labeled with PKH67. Then HUVECs/ Macrophage in co-cultured with labeled SEVs or labeled-SEVs-cells were fixed, stained with Hoechst. Images were obtained using a TCS SP-5 confocal microscope (Leica Microsystems, Wetzlar, Germany). Image J soft was used to analyze uptake efficiency.

### Zebrafish model and microinjection

Zebrafish were raised in the Zebrafish Center at Nantong University Jiangsu Key Laboratory of Neuroregeneration. Zebrafish embryos raised at 28.5 °C through natural mating. To block pigmentation and make embryos development visual, PTU was used at 24 h post-fertilization (hpf). For NPC metastasis analyses, fli1a: EGFP zebrafish embryos at 48hpf were collected. A total 2 g/mL Dil (Beyotime, C1036) was used to label cells. And about 100–500 NPC cells re-suspended in 5 nL medium were injected into the perivitelline space using a microinjection system. Then, zebrafish embryos were transferred into PTU aquarium water and raised at 28.5 °C. Two days after injection, embryos were checked for investigating NPC cell invasion and metastasis using a fluorescent microscope.

### Luciferase assays

Assays were done as previously described [24]. The wild-type M6PR 3′UTR segments or M6PR 3′MUTR containing miRNA-binding site mutations subcloned into psiCHECK-2 vector were transfected into HUVECs with different miR-6750 level using lipofectamine 3000. After 24 h, Dual-Luciferase Report Assay Kit (Promega) was used to measure luciferase activities according to the manufacturer’s directions.

### Mice Animal models: xenografts and SEV conditioning

Animal xenograft models were performed as previously described [47]. Briefly, we established NPC xenografts in 7 to 8-week-old BALB/c nude mice by tail injecting 2 × 10^6^ CNE-2 cells resuspended in 100 µL RPMI 1640 medium. After 5 weeks, the mice were sacrificed and tumour weight (g) was assessed. For analyzing metastasis, lung tissue was removed, fixed, embedded and subjected to IHC.

For SEV injecting, 20 μg of purified SEVs isolated from CNE2-CM were injected into the tail vein 3 day after CNE2 injection. After first injection, SEVs were injected trough the tail vein weekly until sacrificed.

### Statistical analysis

GraphPad Prism® software was applied to conduct statistical analyses. Proper statistical tests were used according to the variance, matching pairs, and distribution. *P* ≤ 0.05 (*), *P* ≤ 0.01 (**), *P* ≤ 0.001 (***) was considered significant. Data were represented as mean ± SEM.

## Supplementary information

Agreement on new author list

Original western blots

Supplymentary materials

## Data Availability

All data needed to evaluate the conclusions in the paper are presented in the paper and/or the Supplementary Materials. Additional data related to this paper may requested from authors.
