# Supplementary figures and images for: SEVs-mediated miR-6750 transfer inhibits pre-metastatic niche formation in nasopharyngeal carcinoma by targeting M6PR
Source: Cell Death Discov. 2023 Jan 6;9:2. doi: 10.1038/s41420-022-01262-4 (PMC9823008; doi:10.1038/s41420-022-01262-4)

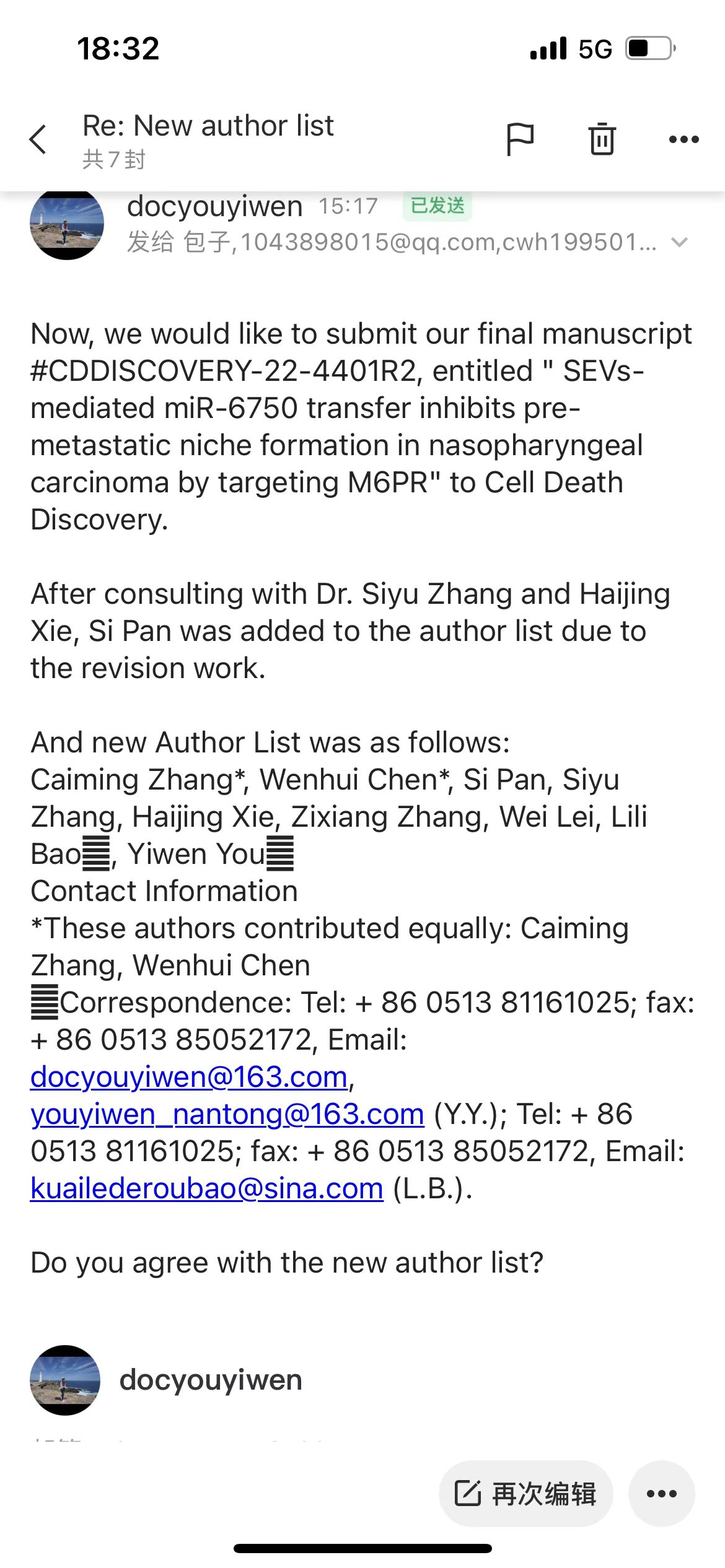


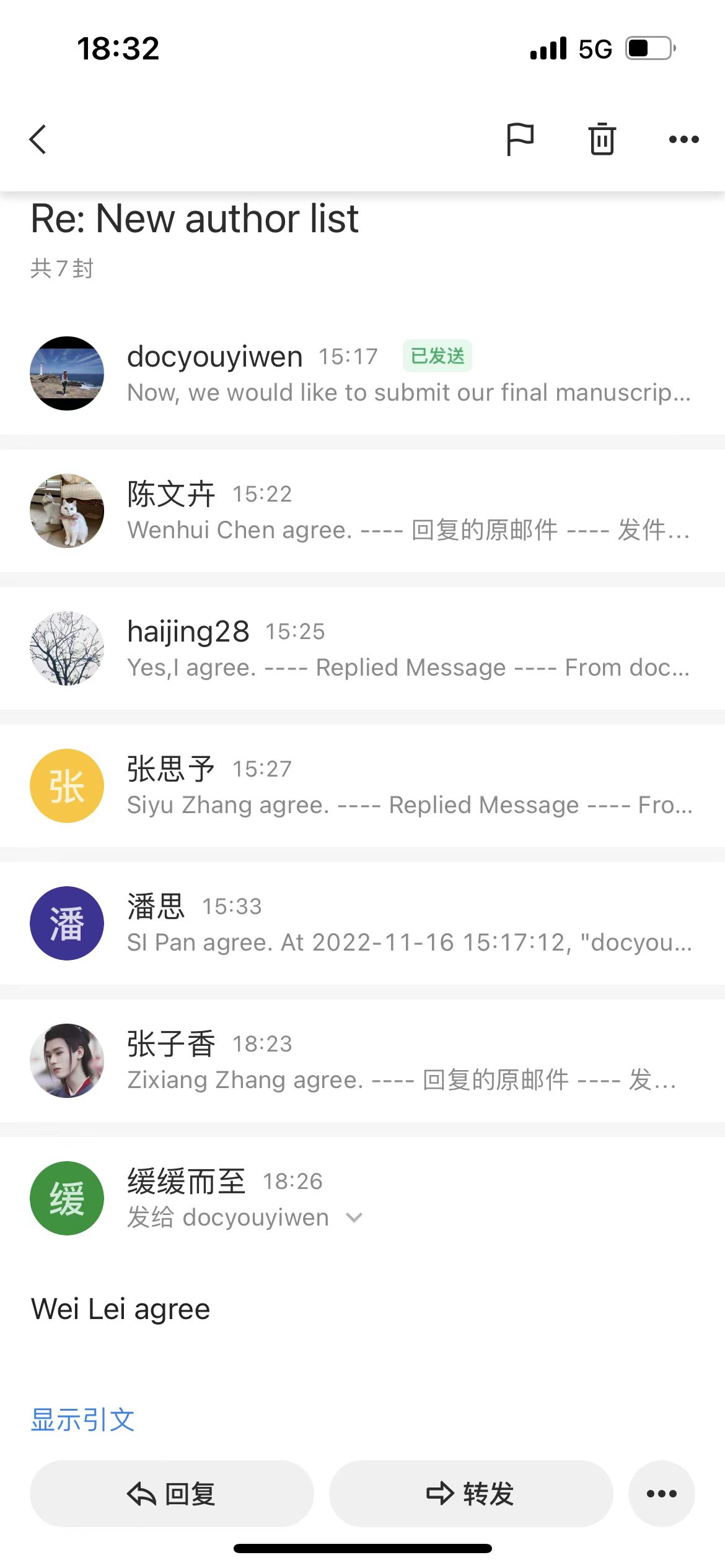


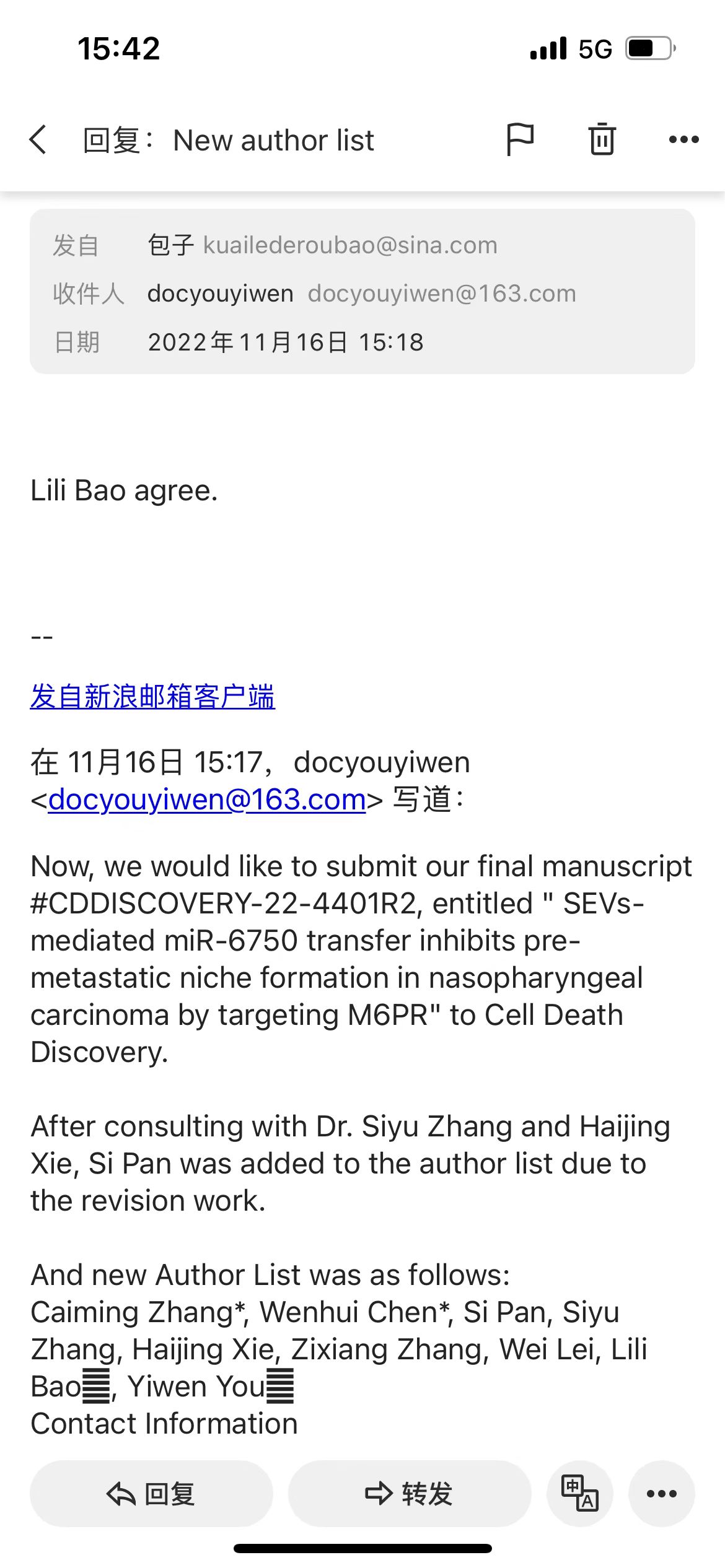


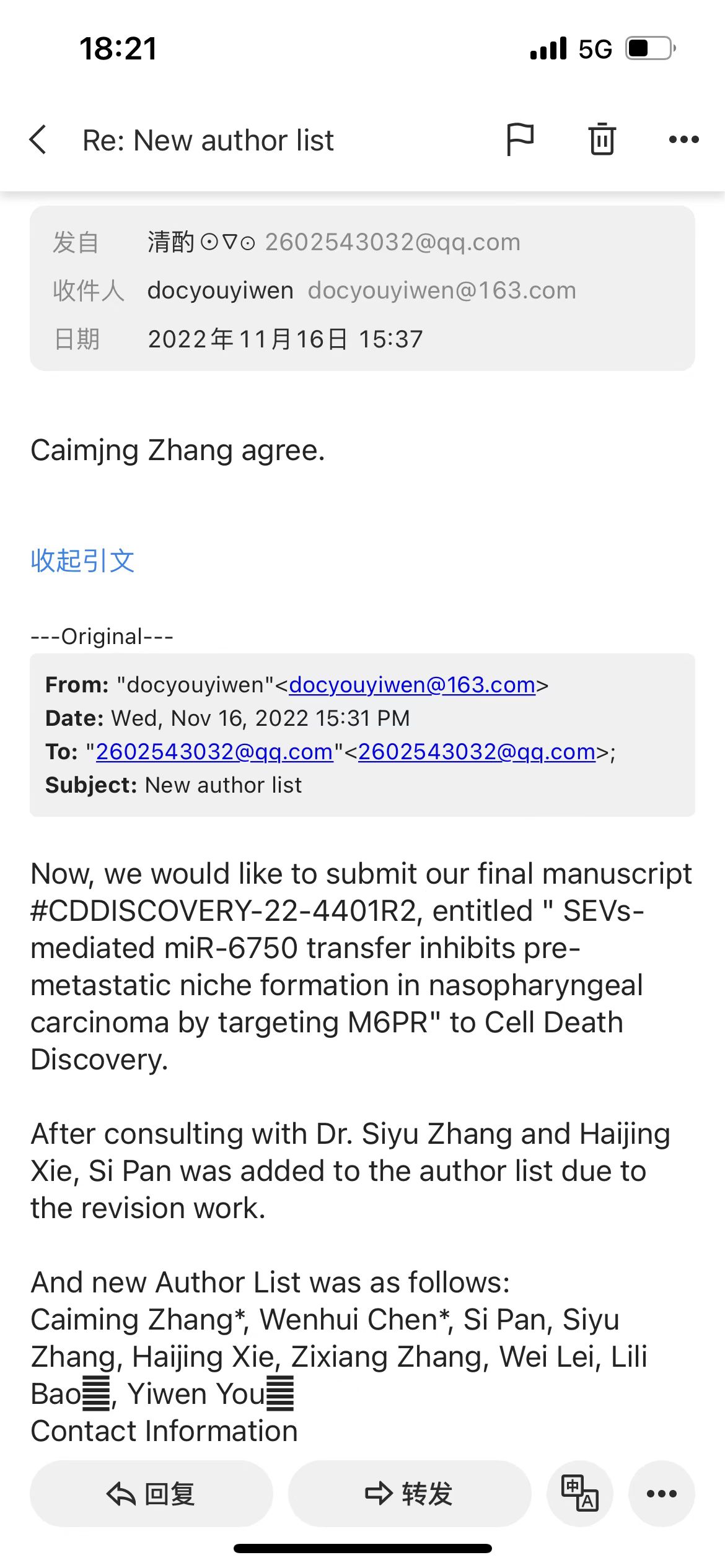

Supplement: Supplementary file 1 — Agreement on new author list [file 41420_2022_1262_MOESM1_ESM.docx]

Figure1A


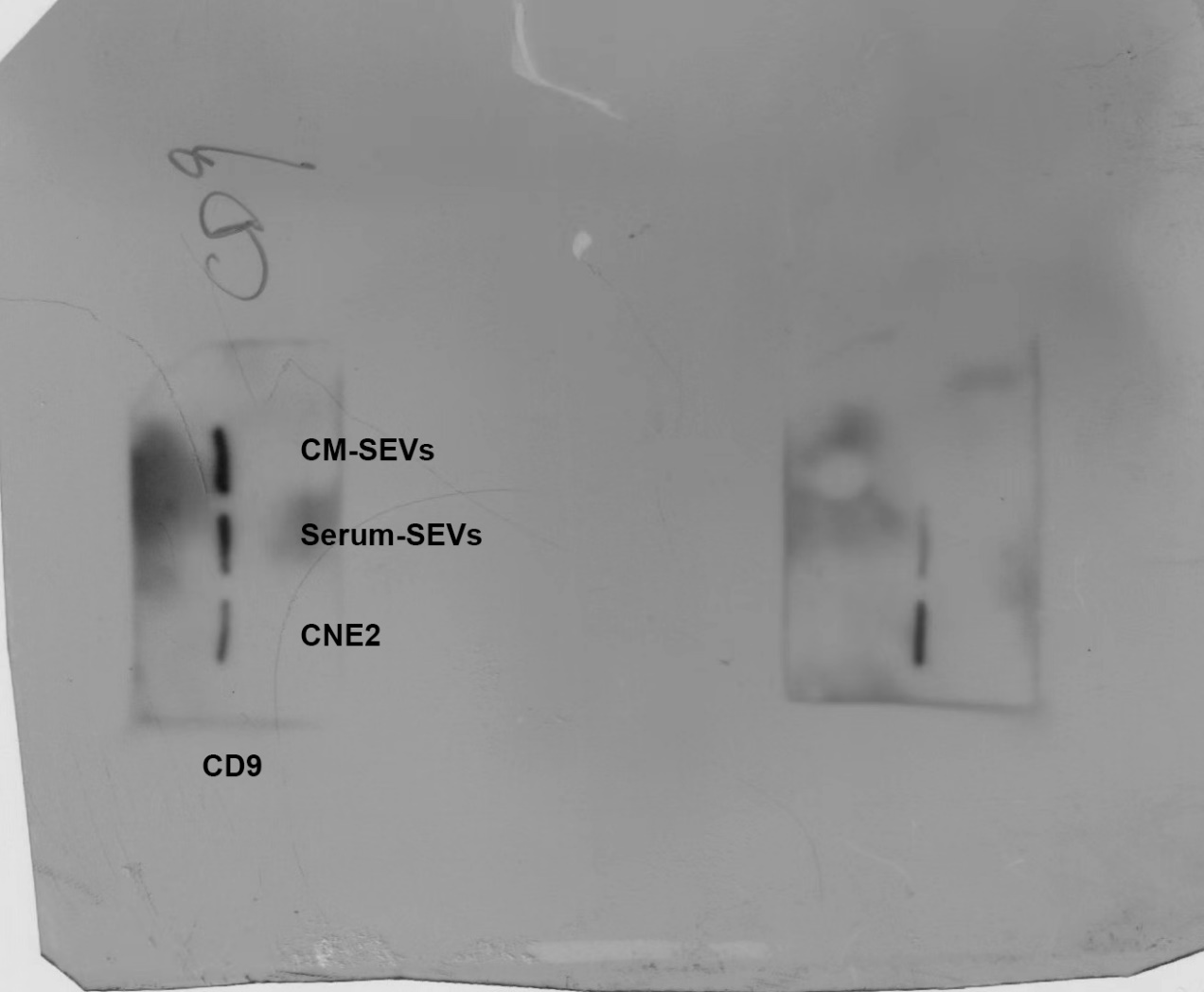

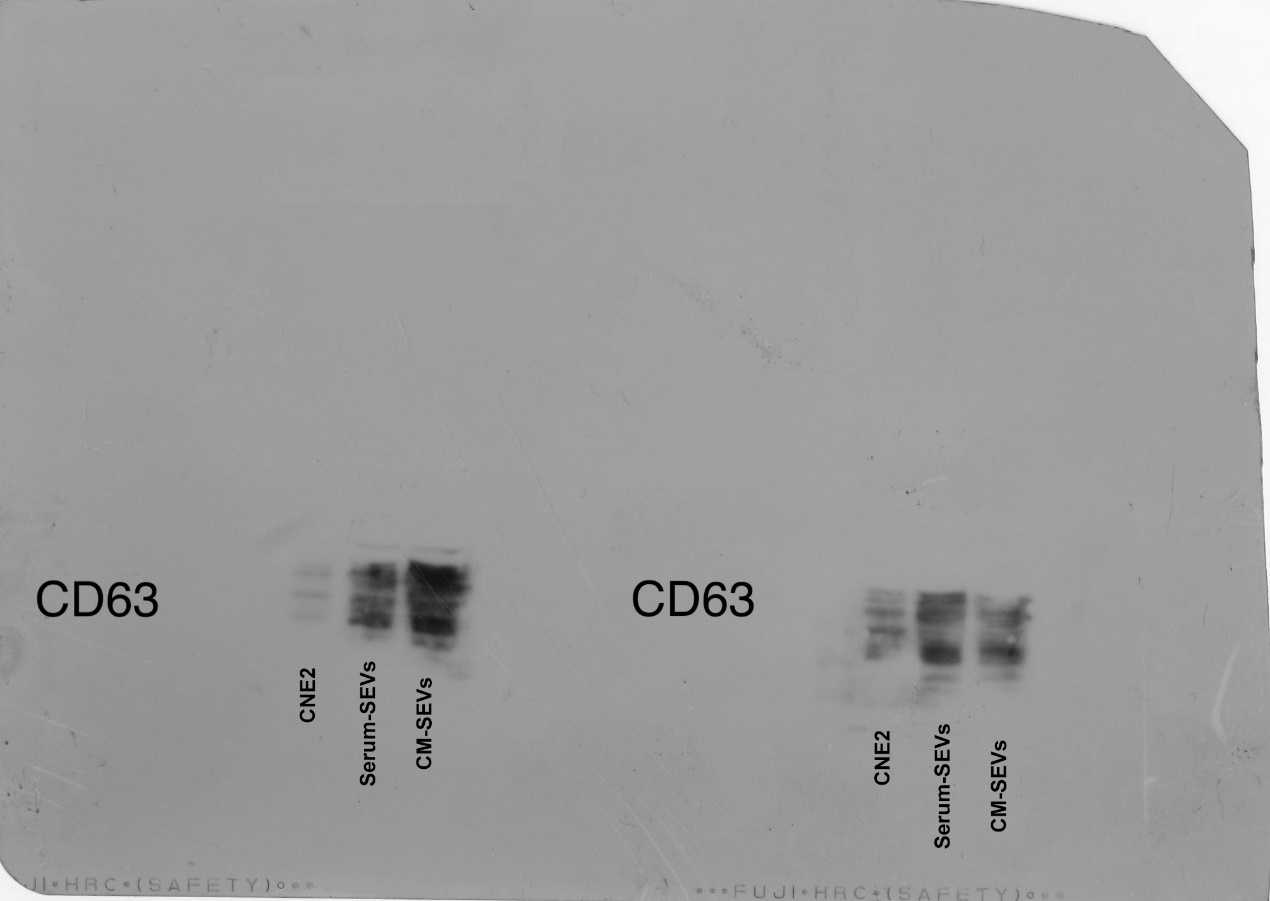

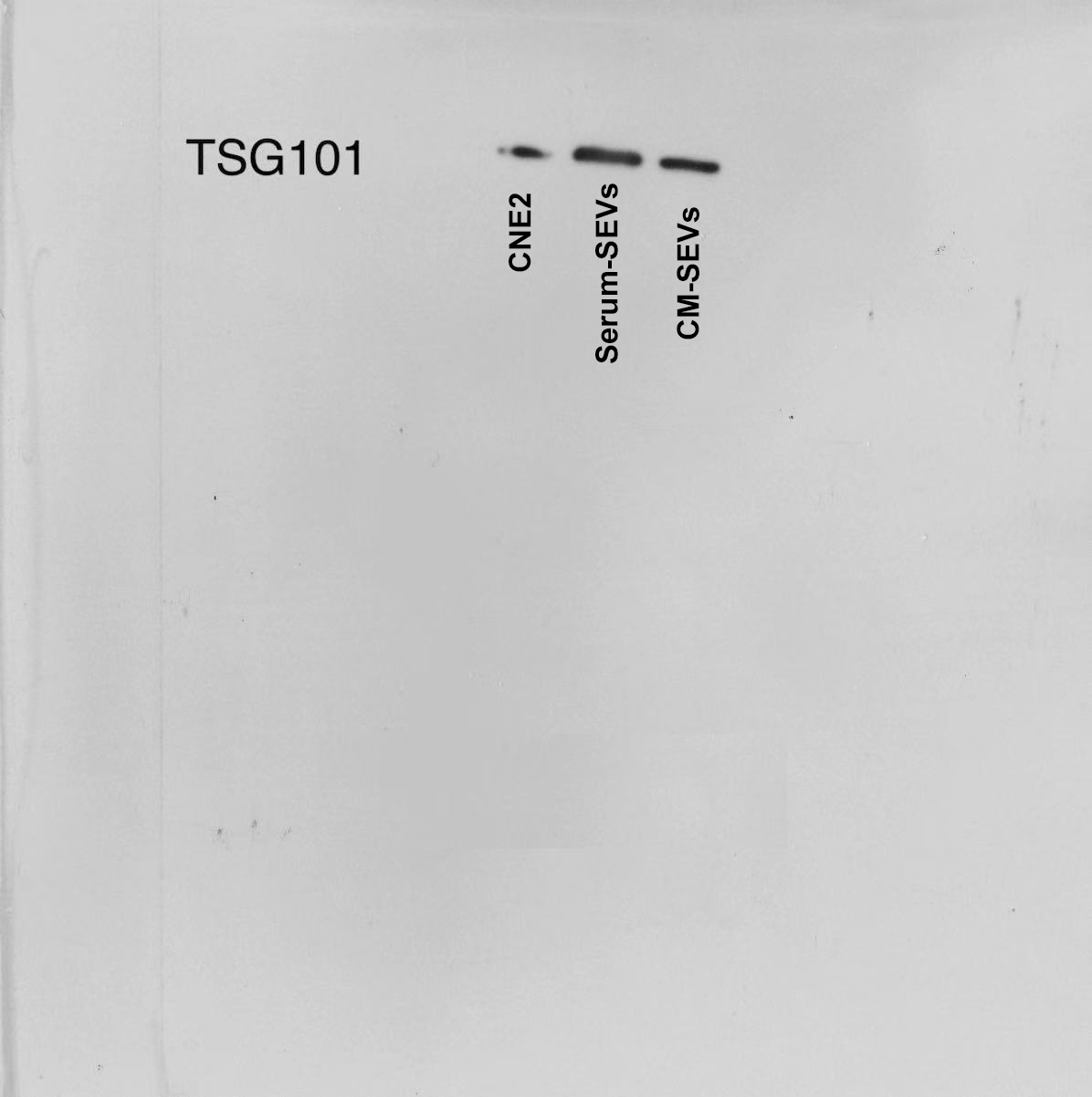

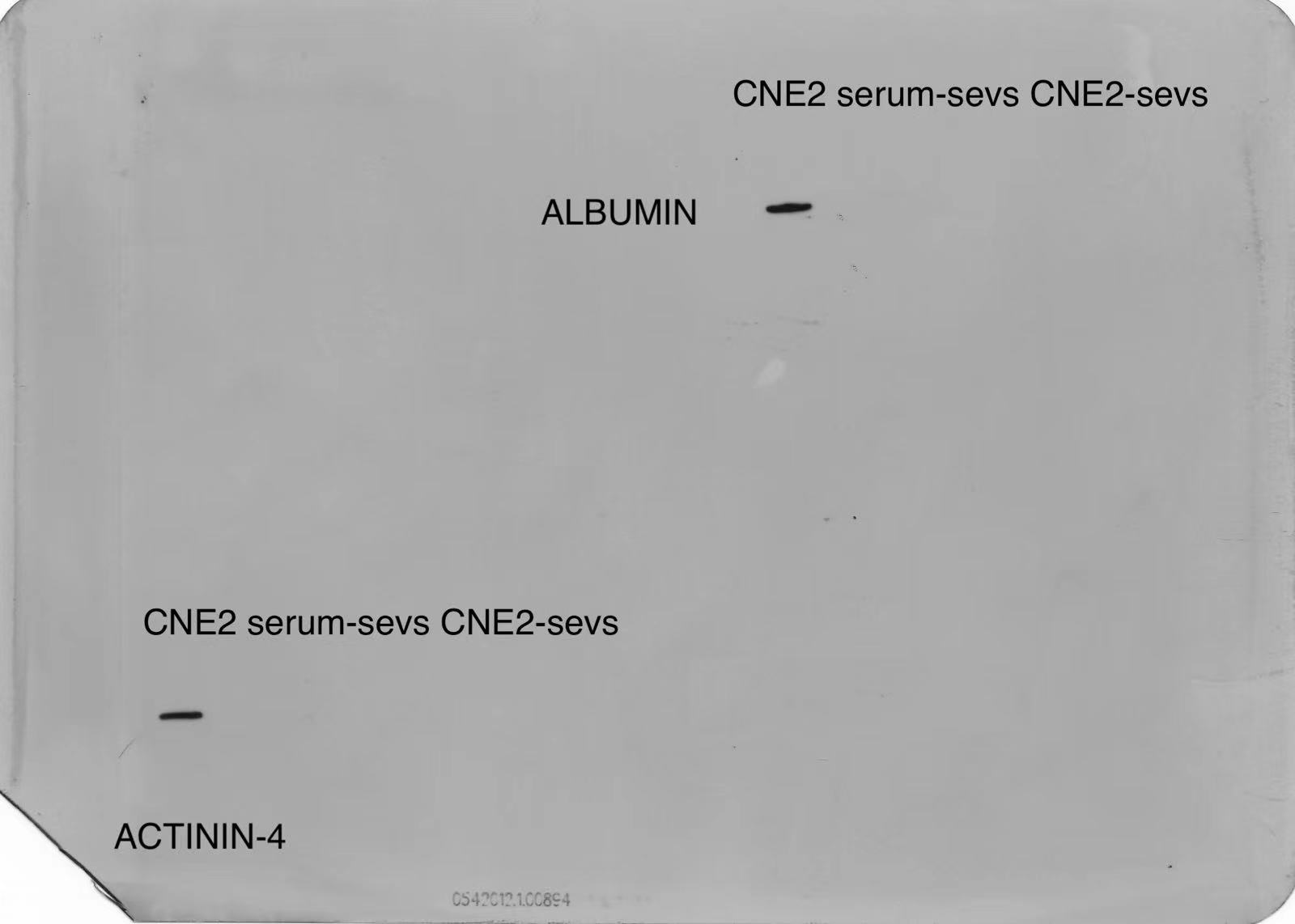

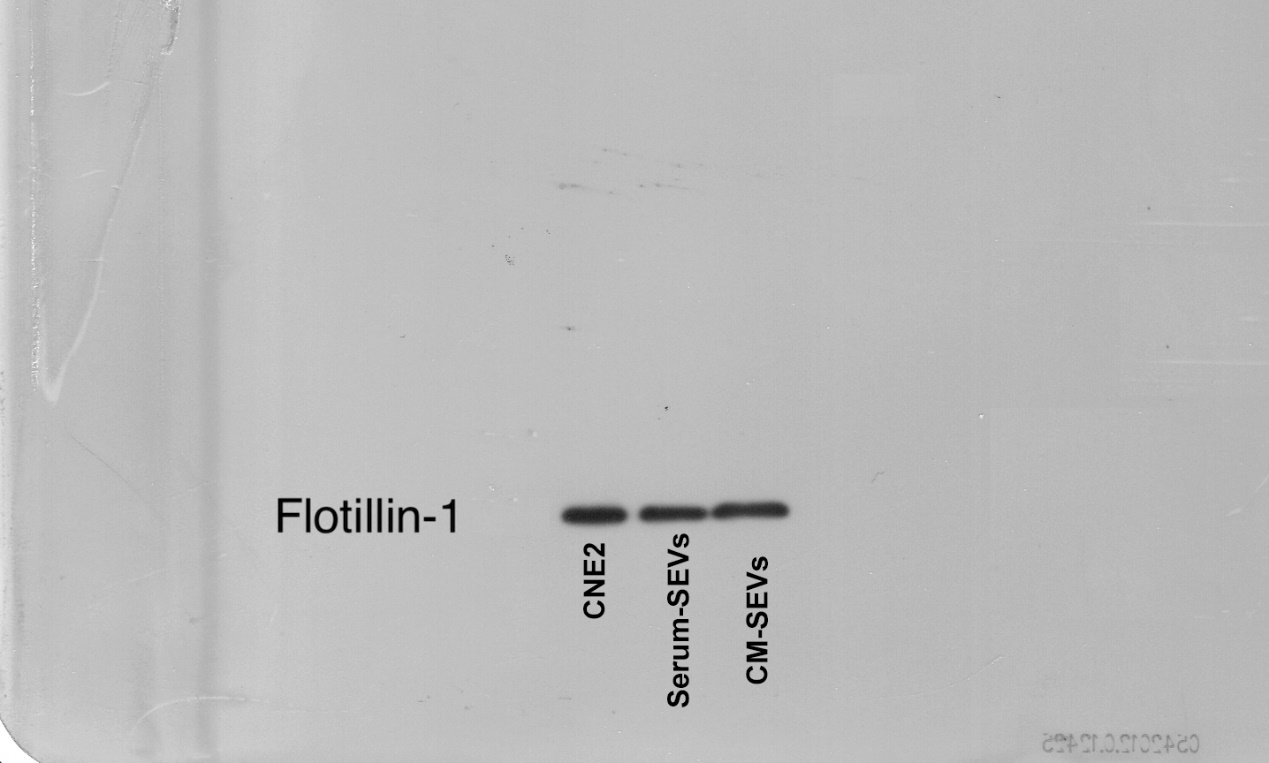


Figure7C


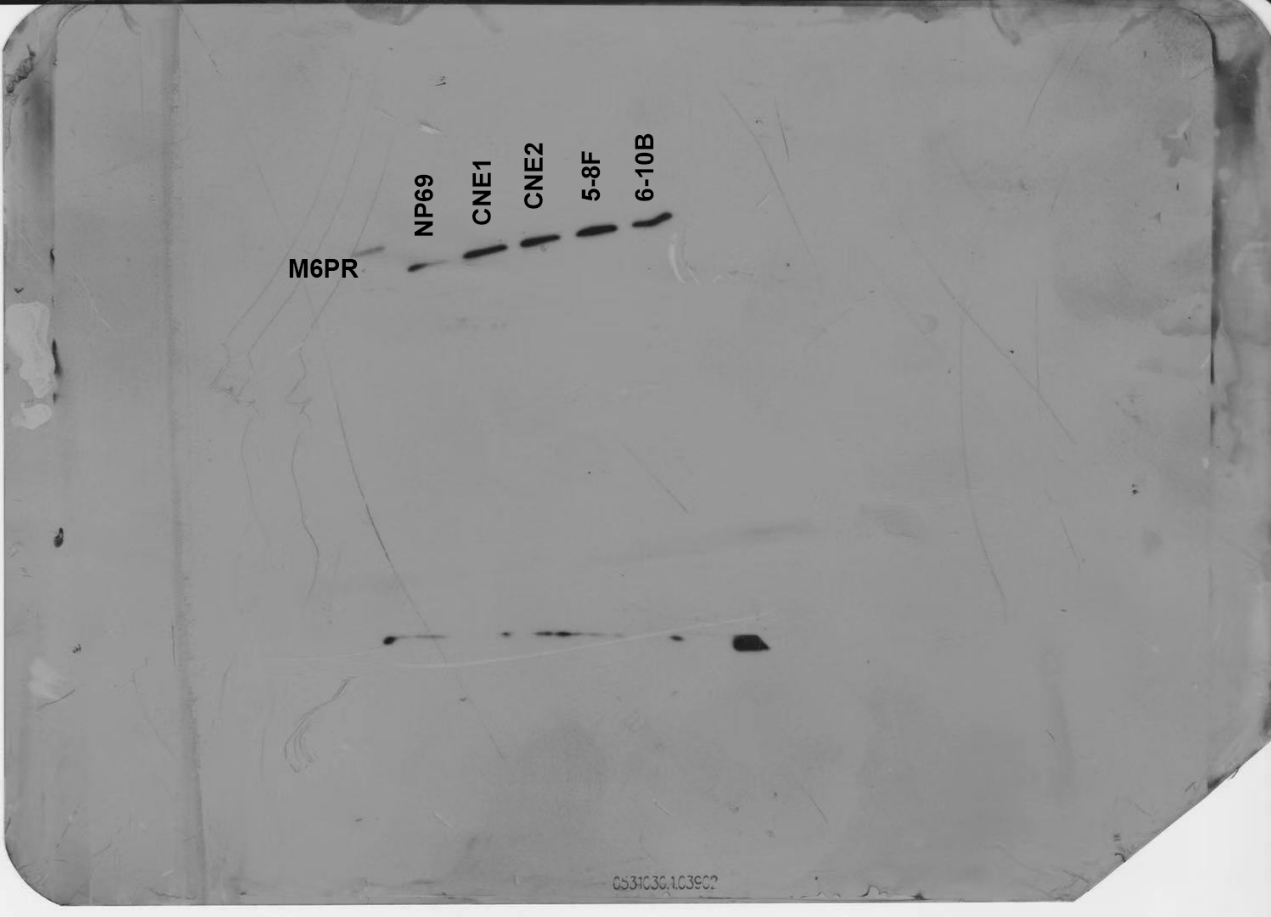


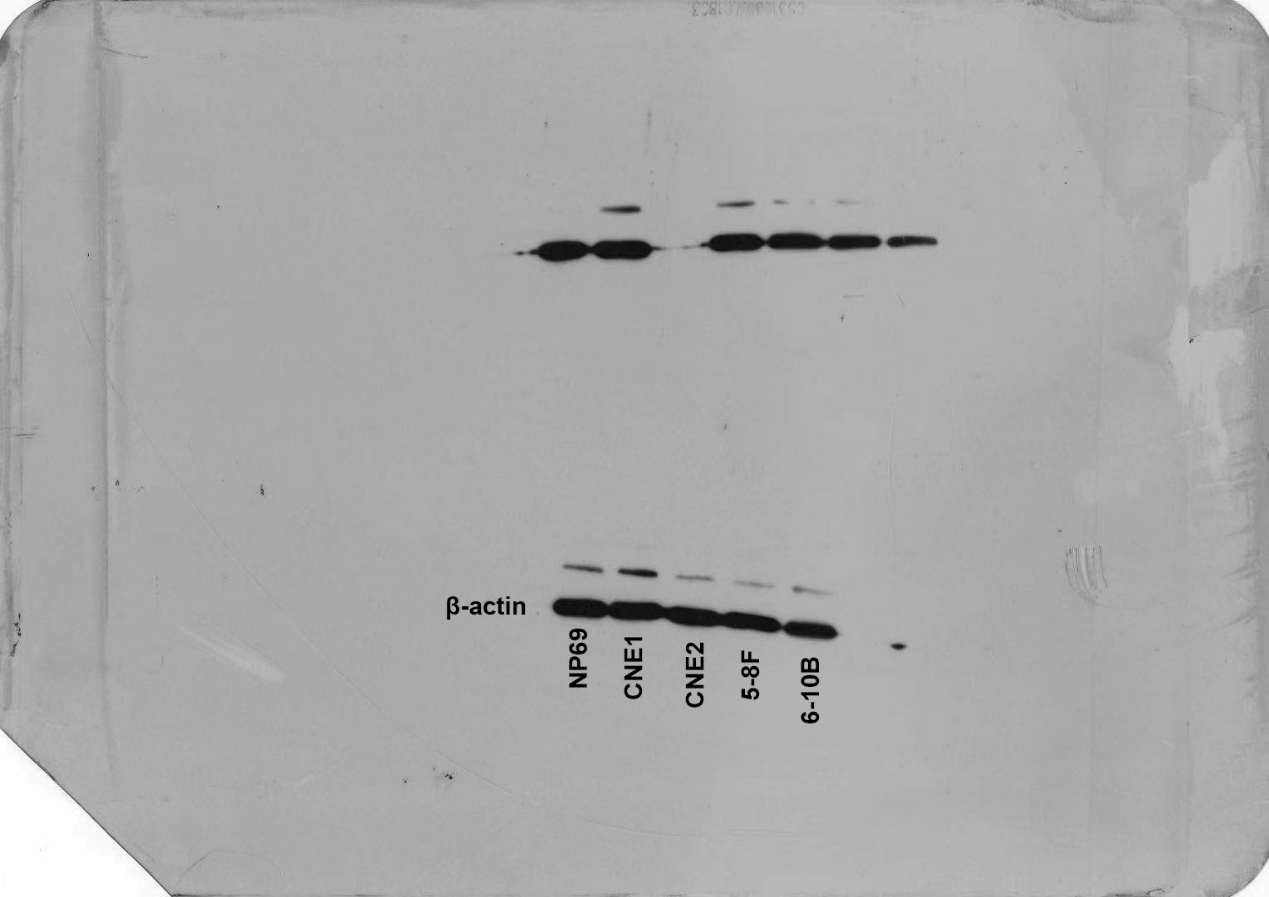


Figure7G


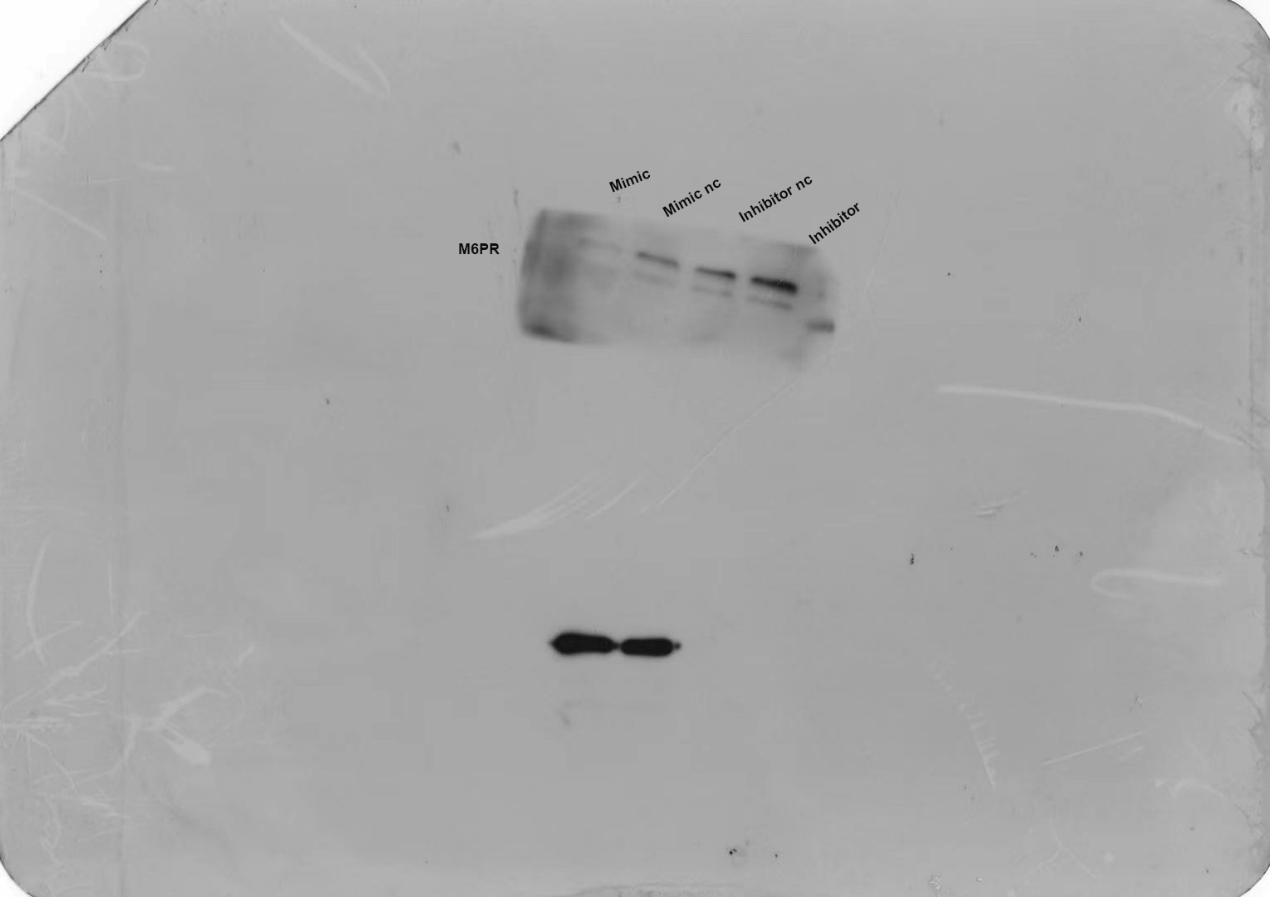





Figure 7I+FigureS4A


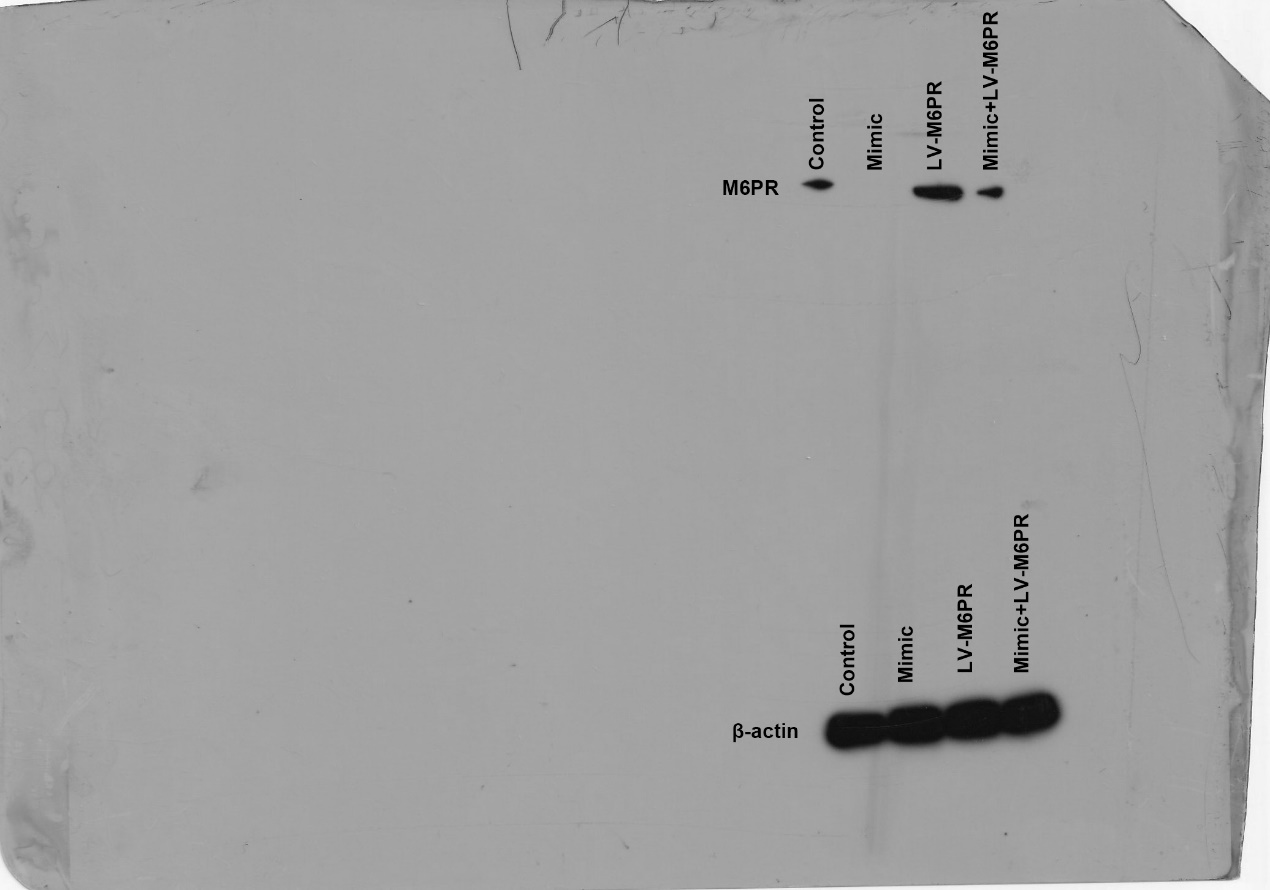


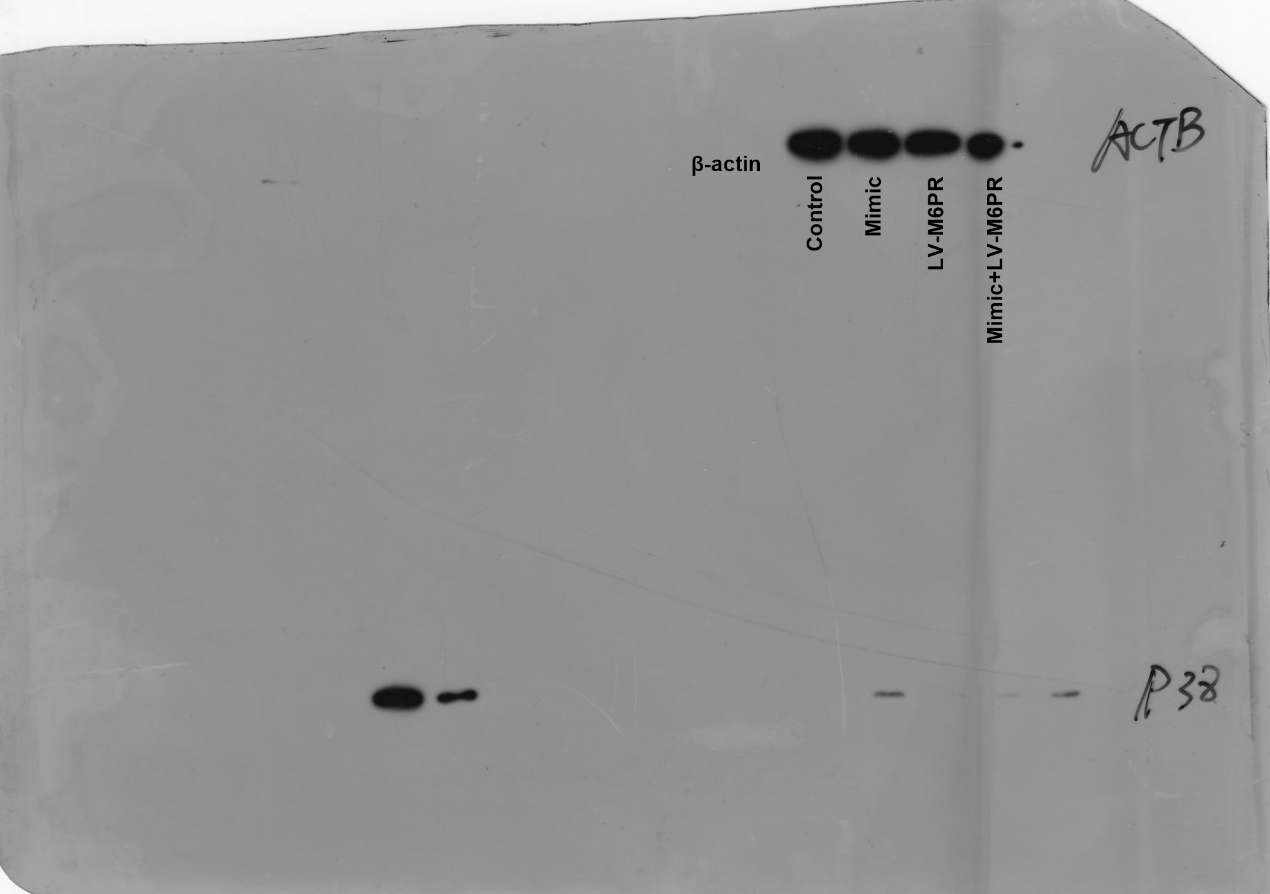


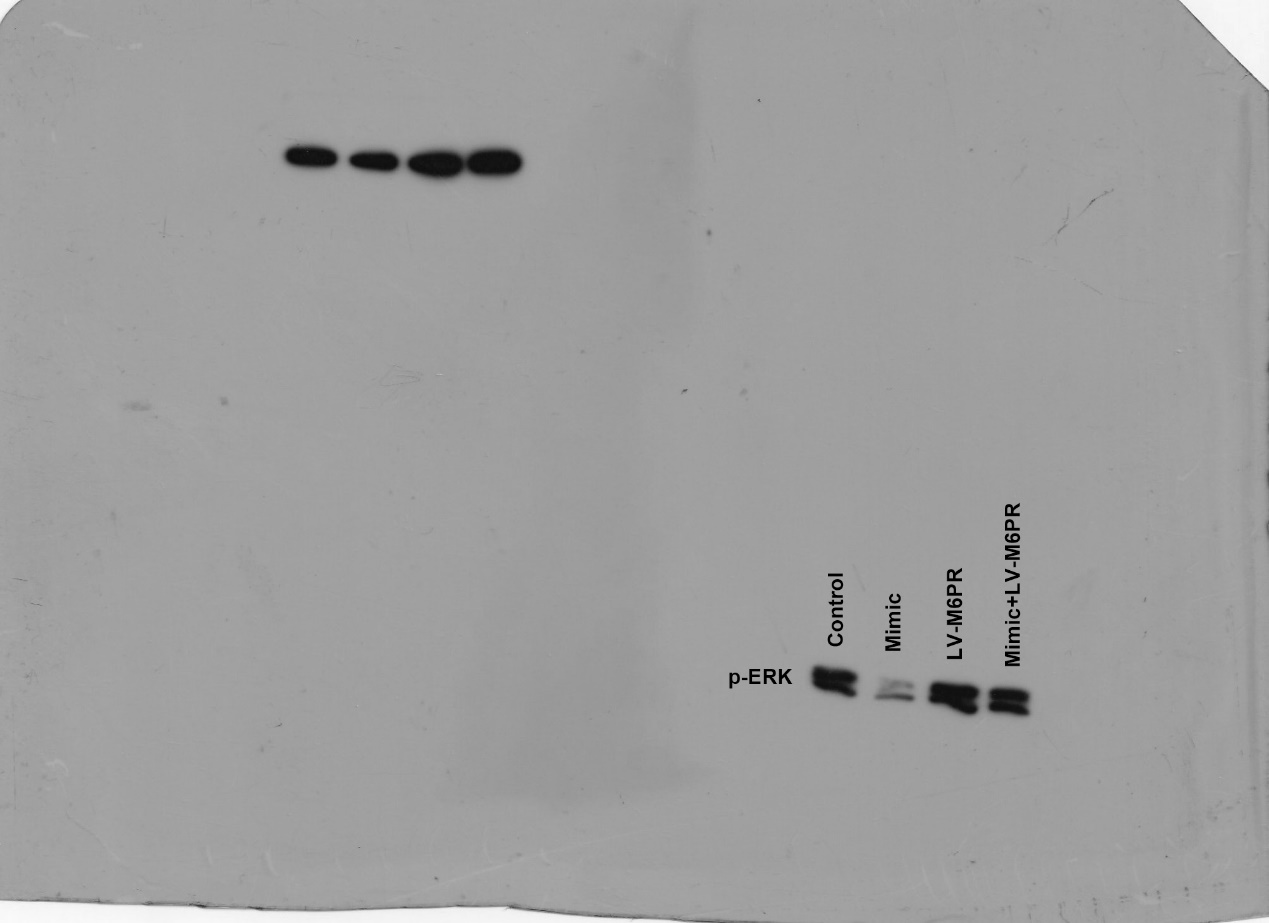


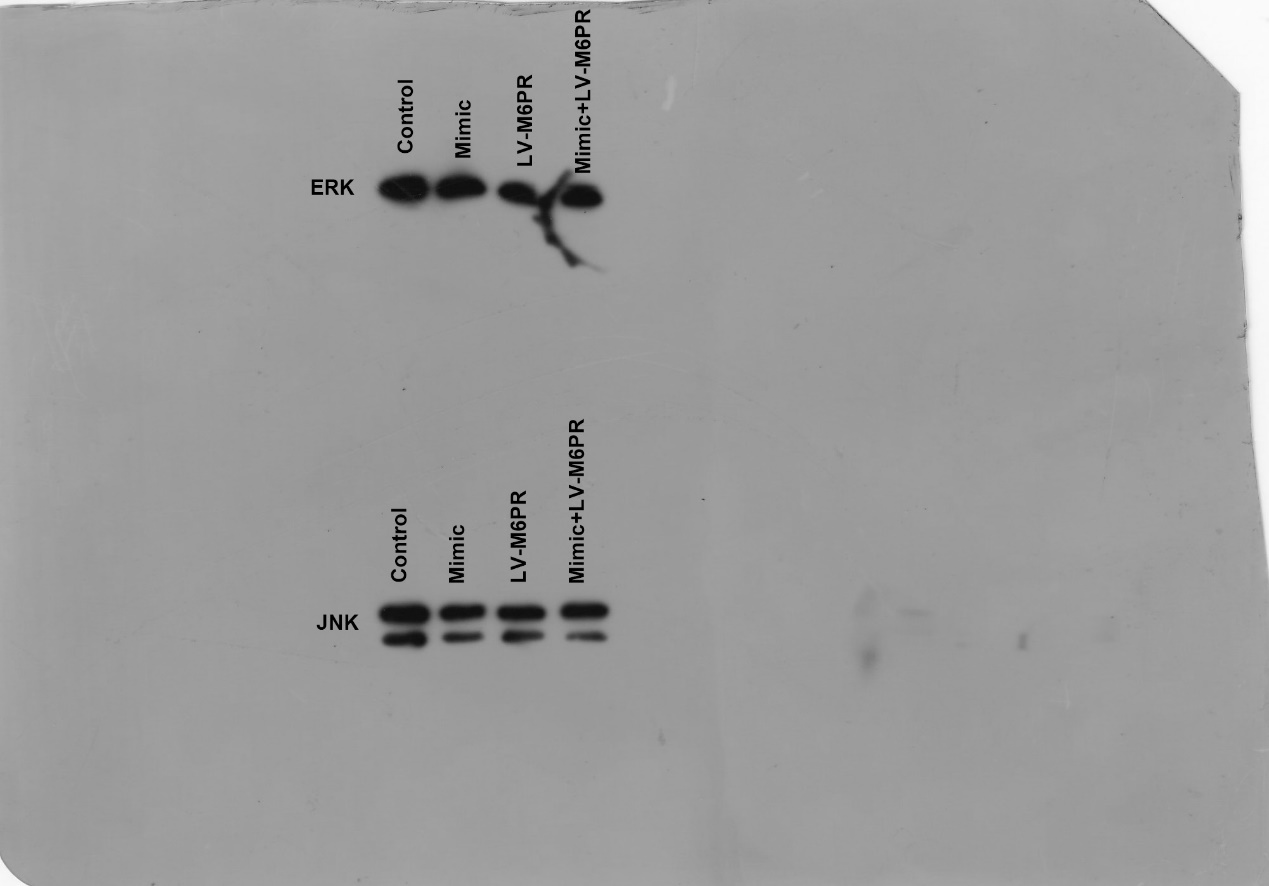


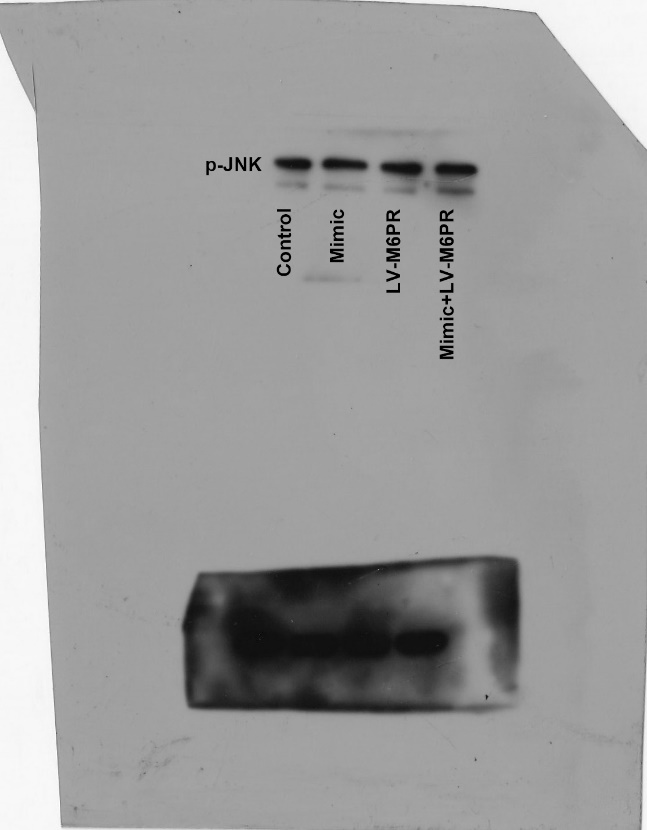

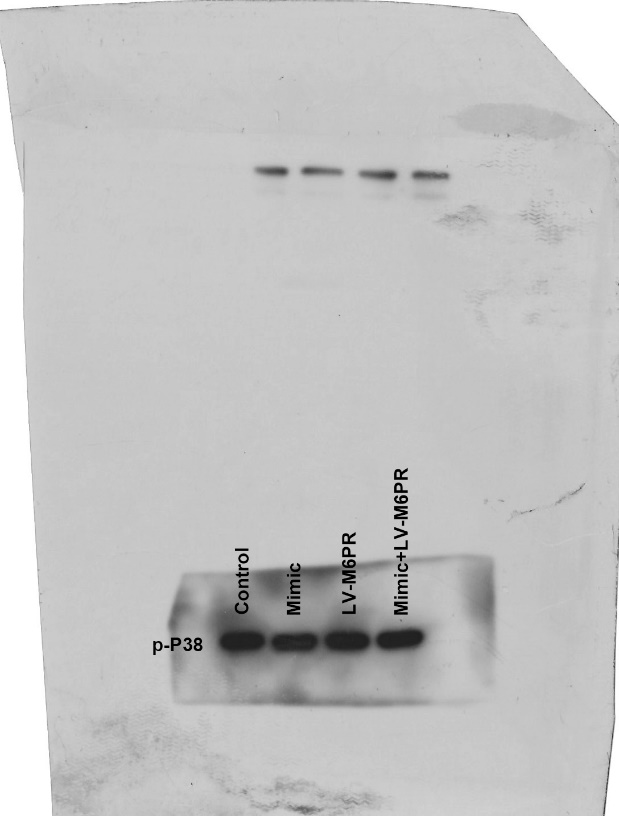

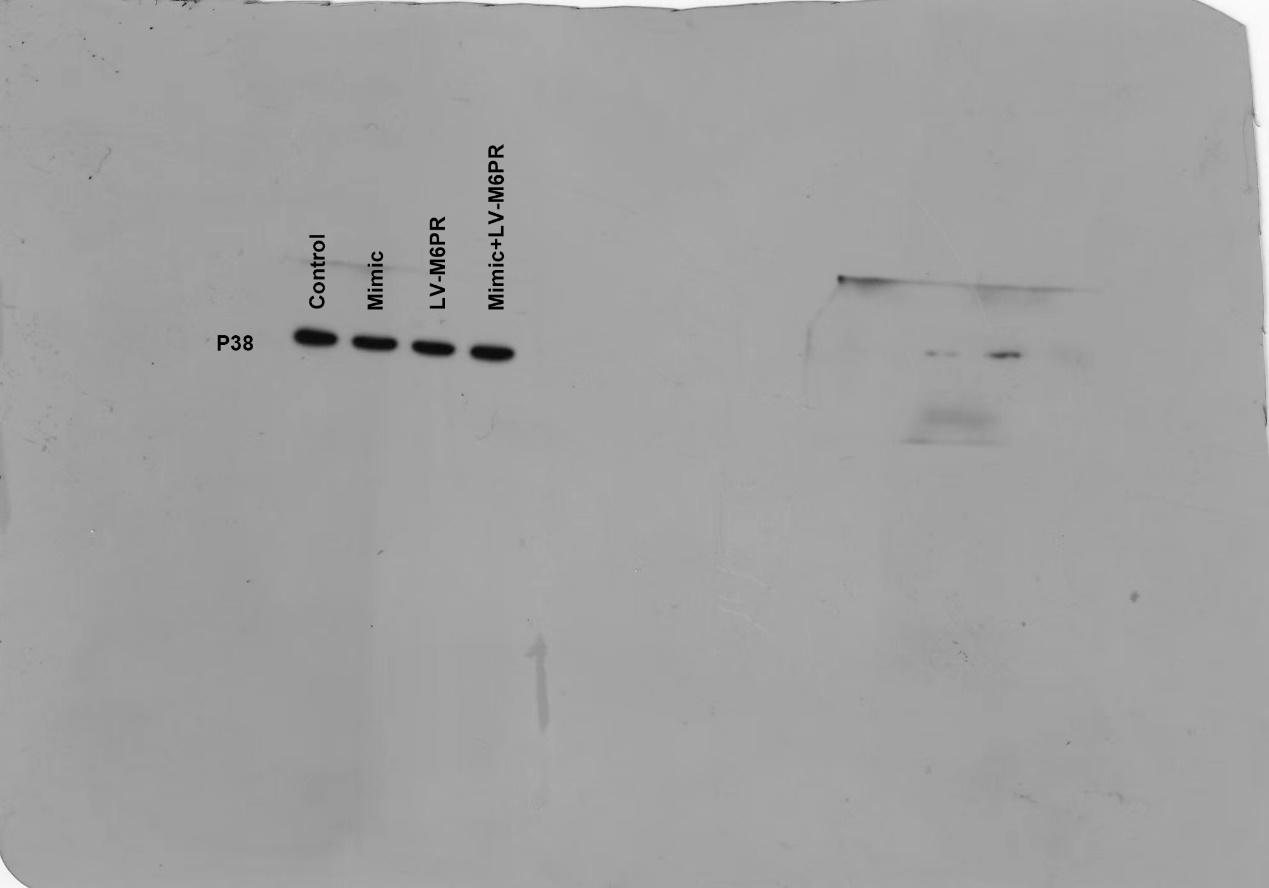


FigureS5B+5D


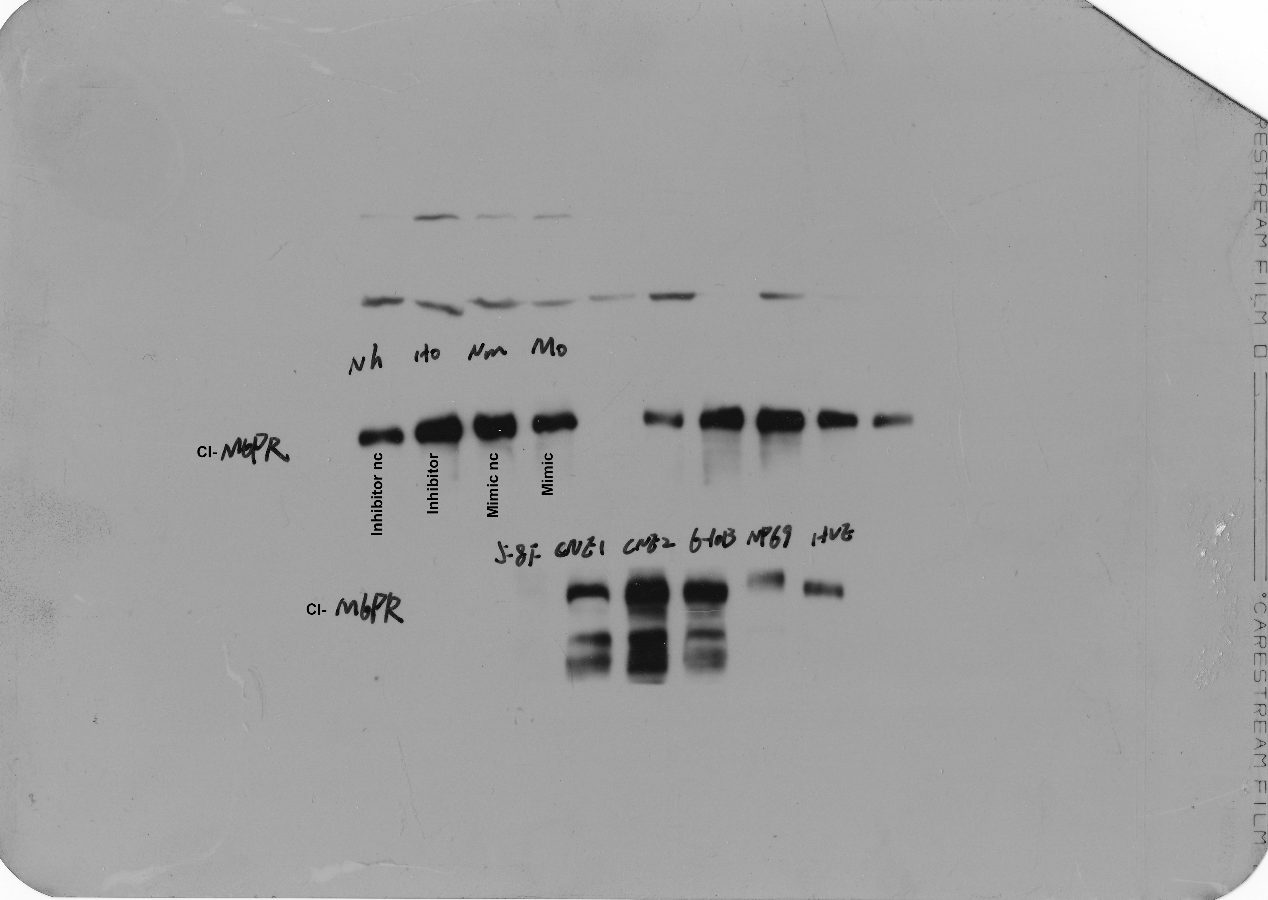




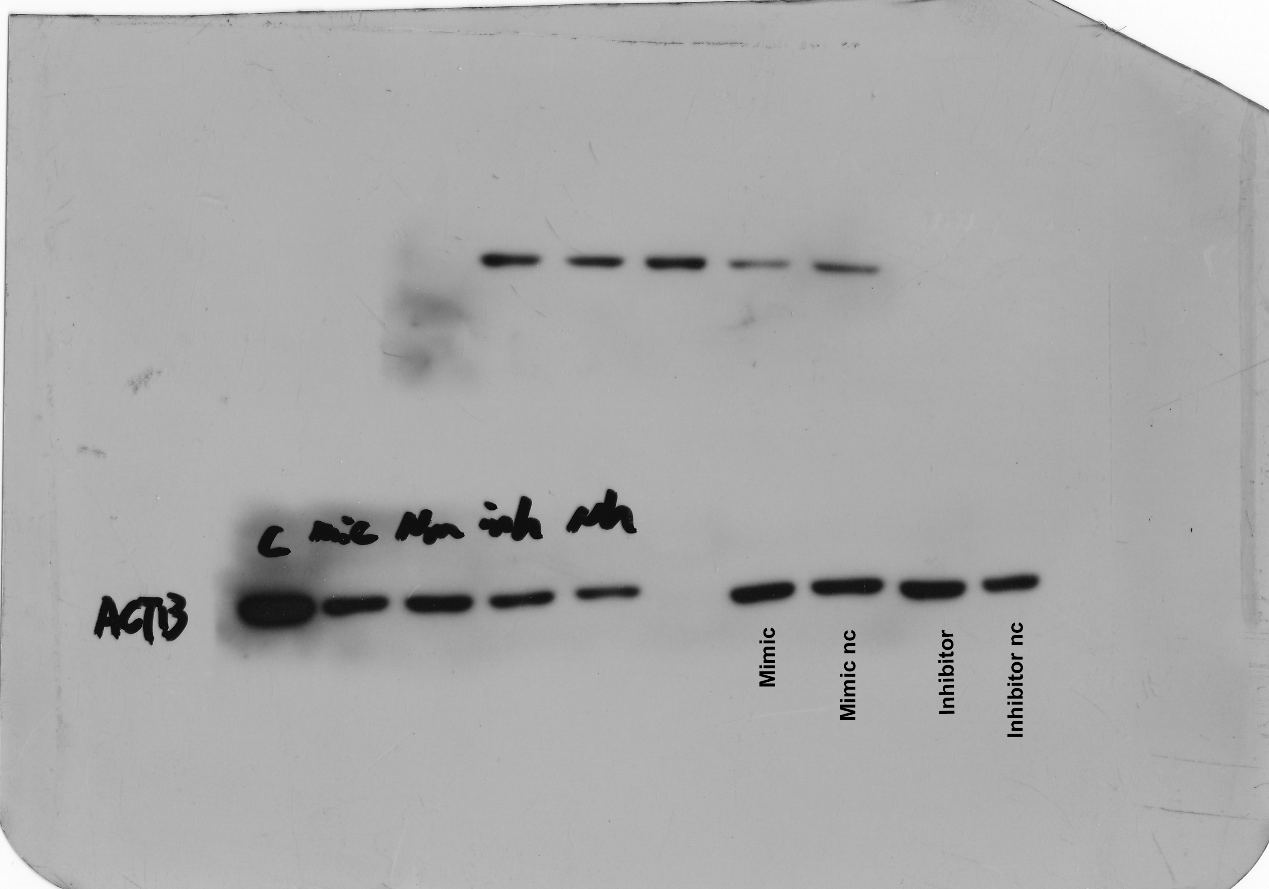

Supplement: Supplementary file 2 — Original western blots [file 41420_2022_1262_MOESM2_ESM.docx]
