# Supplementary material for: SEVs-mediated miR-6750 transfer inhibits pre-metastatic niche formation in nasopharyngeal carcinoma by targeting M6PR
Source: Cell Death Discov. 2023 Jan 6;9:2. doi: 10.1038/s41420-022-01262-4 (PMC9823008; doi:10.1038/s41420-022-01262-4)
Supplement: Supplementary file 3 — Supplymentary materials [file 41420_2022_1262_MOESM3_ESM.docx]

**Supplymental information**


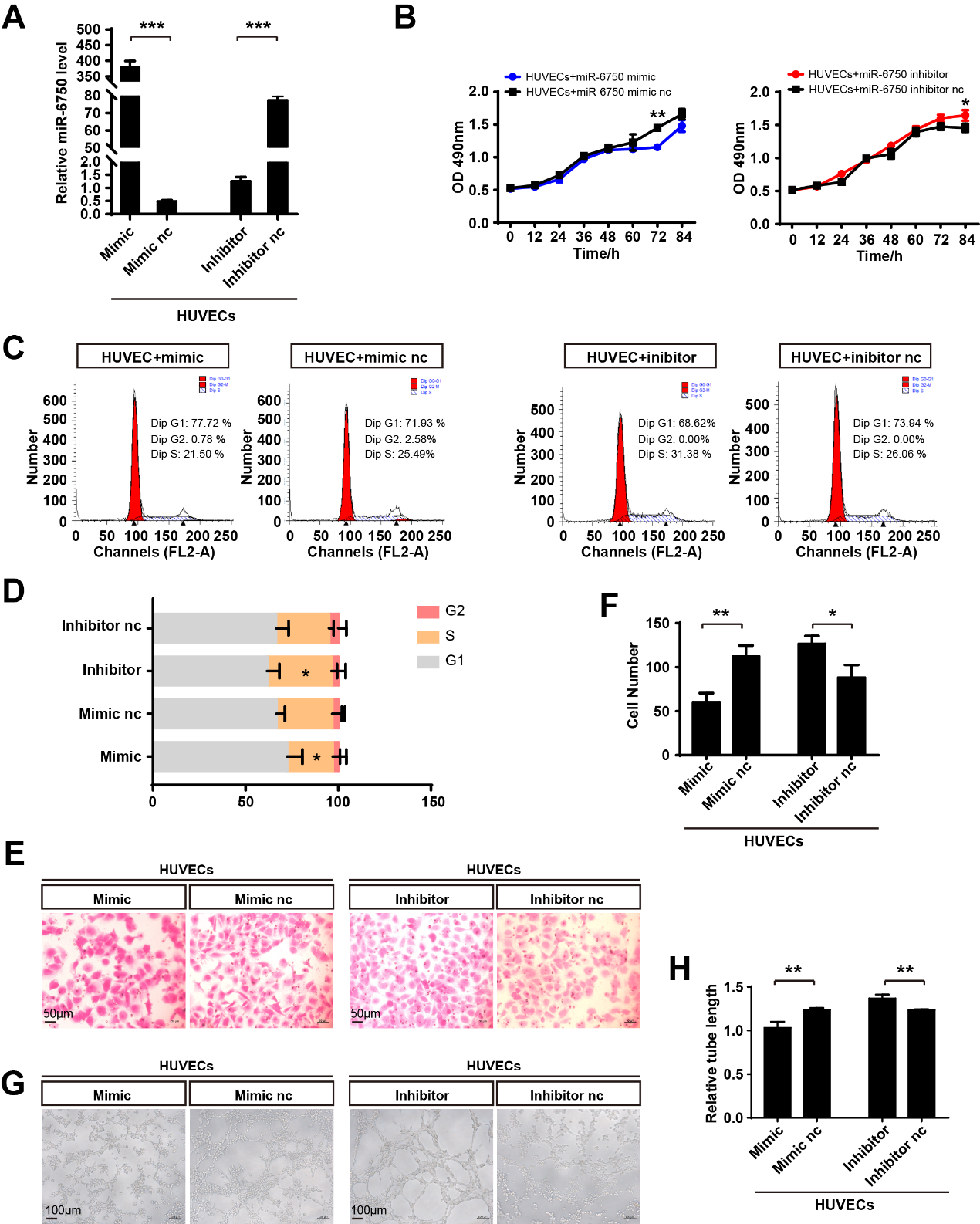


**FigureS1 MiR-6750 regulated *in vitro* angiogenesis.** (a) Transfection efficiency of miR-6750 was calculated. Student’s t-test. (b) CCK8 assay was performed to measure viabilities of HUVECs treated as graph-depicted groups. Two-way ANOVA. (c-d) Cell-cycle analysis was used. One-way ANOVA. (e-f) Transwell migration assays were performed to measure cell migration. Student’s t-test. (g) Tube formation assays were performed using Matrigel. Student’s t-test.


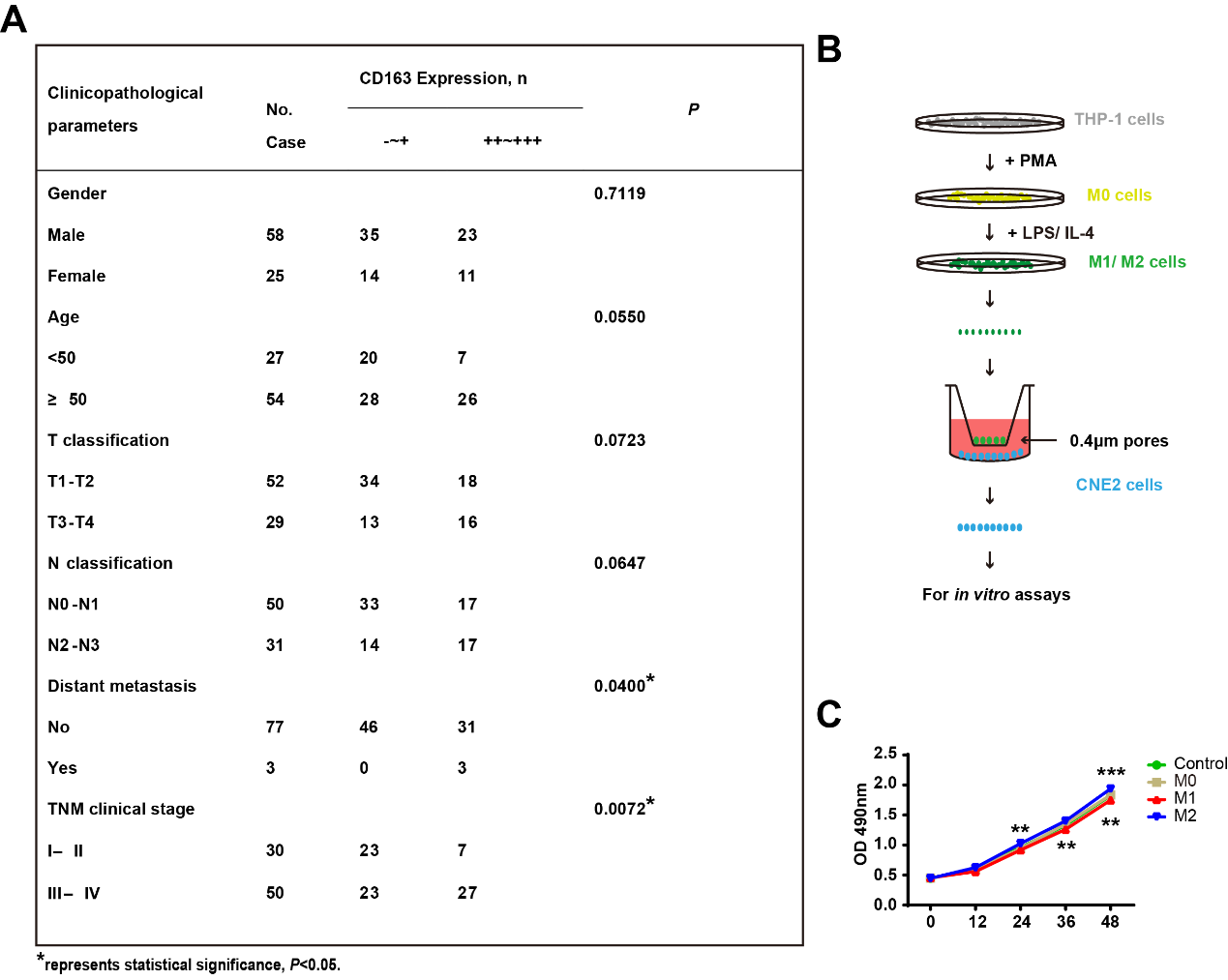


**FigureS2 Clinical significance of M2 macrophage in NPC. (a)** Correlation between clinicopathologic parameters and CD163 level. (b) Scheme of CNE2 cell collection after co-cultured with macrophage. (c) The cell growth of CNE2 treated as graph-depicted groups was measured by CCK8 assay. Two-way ANOVA.


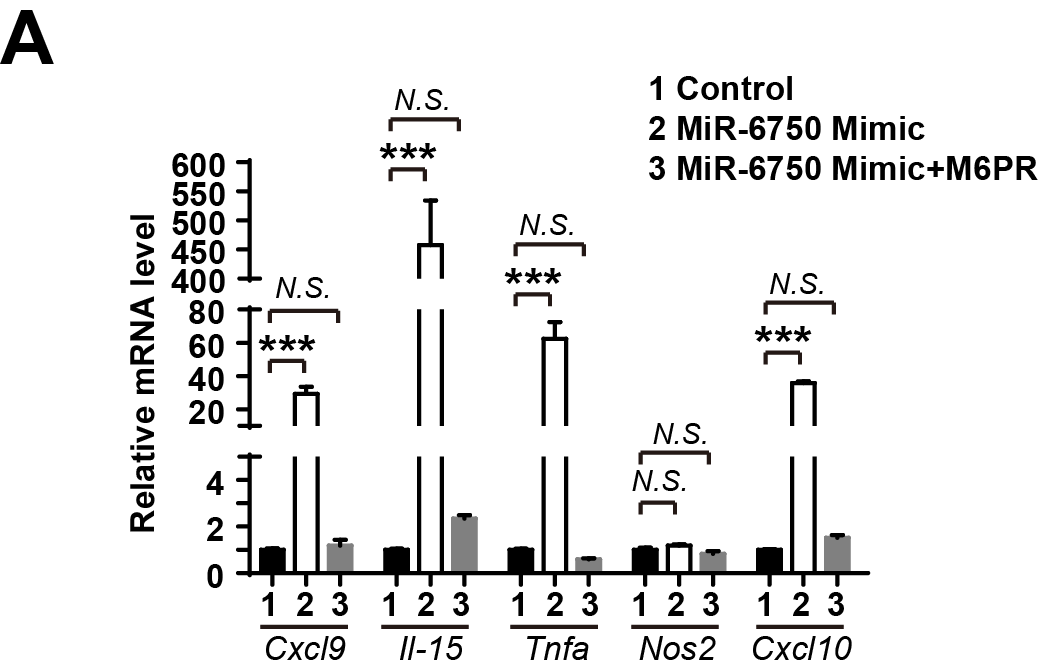


**FigureS3 MiR-6750 regulates macrophage phenotype through the target molecule M6PR**. (a) M0 Macrophages treated as graph-depicted groups was used to measure Macrophage phenotype by qRT-PCR assay. Student’s t-test.


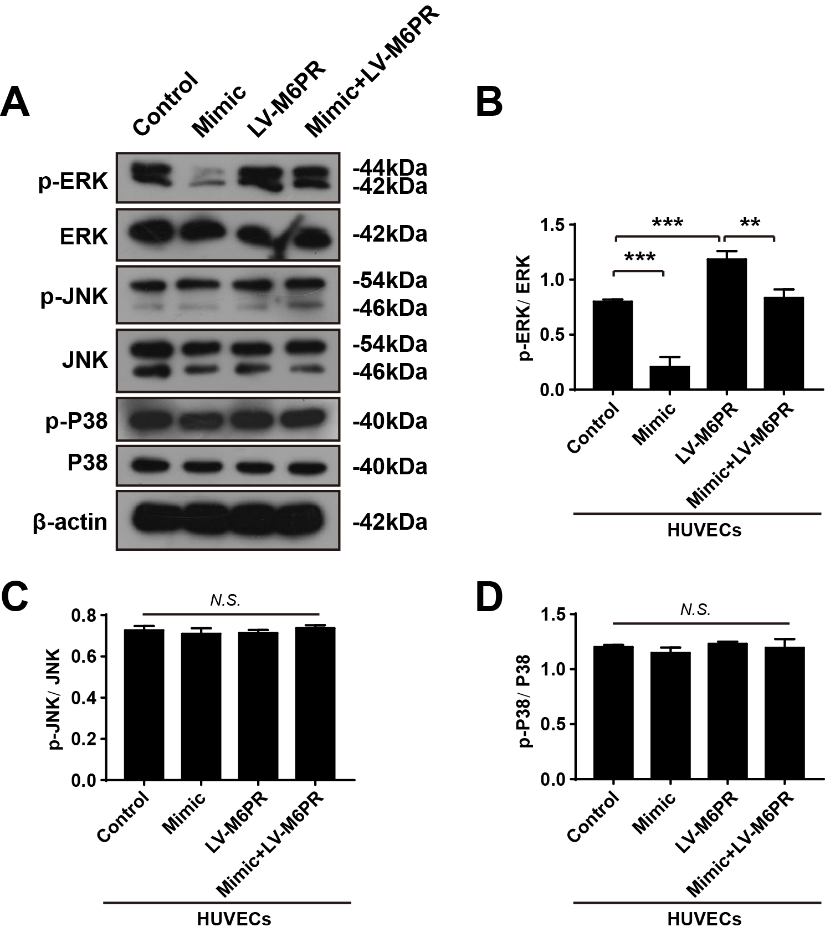


**FigureS4 MiR-6750-M6PR axis regulate MAPK pathway.** (a-d) Western blot of p-ERK, p-JNK and p-P38 in HUVECs treated as indicated. The results were analyzed with ImageJ software. One-way ANOVA.


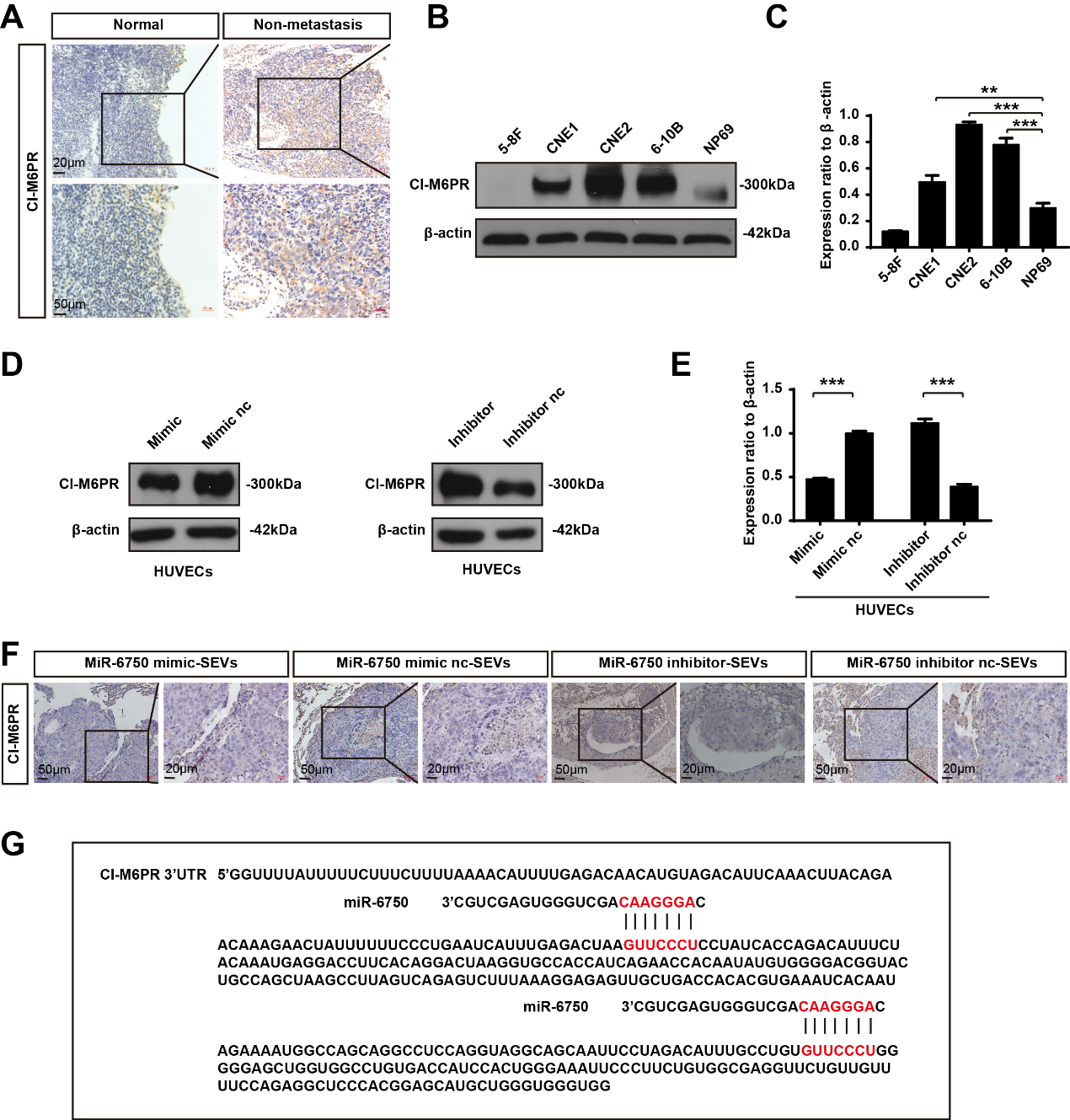


**FigureS5 The potential relationship between miR-6750 and CI-M6PR.** (a) Representative images of HE staining. (b-c) Levels of CI-M6PR in NPC cells measured by western blot. Student’s t-test. (d-e) M6PR immunoblotting in HUVECs treated as indicated was analyzed. Student’s t-test. (f) Representative images of CI-M6PR in metastatic lung tissues collected from graph-depicted groups in mice. (g) The binding sites of hsa-miR-6750 and M6PR-3′-UTR.
